# Development and validation of deprescribing algorithms for kidney failure using consensus development methodology

**DOI:** 10.1080/20523211.2026.2666250

**Published:** 2026-05-20

**Authors:** Amani Zidan, Kheloud Awad, Safeya Habib, Noor Alsalemi, Hager Elgeed, Abdullah Hamad, Hassan Al-Malki, Mohamad Alkadi, Mohamed Elesnawi, Fatima Babiker, Rania Ibrahim, Muhammad Abdul Hadi, Ahmed Awaisu

**Affiliations:** a QU-Health, Qatar University, Doha, Qatar; b Pharmacy Department, Hamad Medical Corporation, Doha, Qatar; c College of Pharmacy, QU-Health, Qatar University, Doha, Qatar; d Division of Nephrology, Department of Medicine, Hamad Medical Corporation, Doha, Qatar

**Keywords:** Deprescribing, inappropriate polypharmacy, chronic kidney disease, consensus methodology, guidelines, algorithms, tools

## Abstract

**Background:**

Patients with kidney failure (KF) are at high risk of inappropriate polypharmacy; however, this can be mitigated through deprescribing. While several evidence-based deprescribing guidelines exist for various patient populations, their applicability and utility in KF settings remain limited.

**Aim:**

This study aimed to adapt existing evidence on deprescribing in KF to develop practical deprescribing algorithms that support clinicians in implementing deprescribing interventions in KF care.

**Methods:**

Available evidence about deprescribing in KF was reviewed, and local prescribing trends among KF patients were explored. Second, a panel of five experts in pharmacotherapy, medication safety, and clinical research was convened and conducted consensus development workshops to appraise the evidence and develop comprehensive deprescribing algorithms for patients with KF. The adapted algorithms were reviewed by nephrology consultants (*n* = 3) and clinical pharmacists (*n* = 2) for clinical validation. The validated algorithms were also applied in a pilot deprescribing study to assess their feasibility and utility in this setting.

**Results:**

One general and 18 drug class-specific evidence-based deprescribing algorithms in KF care were developed. These provide a structured, stepwise approach to assessing medication appropriateness and guiding deprescribing decisions. Expert validation resulted in refinements of the algorithms. In the pilot testing among hemodialysis (HD) and low clearance clinics, the generic deprescribing algorithm was applied in all cases. The drug-class-specific algorithms were used in 41 deprescribing plans (55.4%) in the HD and in 5 (45.5%) in the low-clearance clinic settings.

**Conclusion:**

We developed and validated a set of evidence-based deprescribing algorithms tailored to patients with KF, offering a practical and structured approach to support clinicians in deprescribing potentially inappropriate medications.

## Background

Patients with chronic kidney disease (CKD), especially those with kidney failure (KF), experience one of the highest burdens of polypharmacy among chronic disease populations (Liu et al., 2024; Lefebvre et al., 2020). This burden is due to the high prevalence of comorbidities and CKD-associated complications, such as anaemia, mineral bone diseases (MBD), electrolyte imbalances, infections, thyroid disorders, hypertension, diabetes, and cardiovascular diseases (CVDs) (Fraser et al., 2015). Given the progressive nature of the disease, the burden of polypharmacy consequently increases (Battistella et al., 2025), resulting in inappropriate dosing, drug–drug interactions, and drug-disease interactions (Triantafylidis et al., 2018). Studies have reported rates of potentially inappropriate medications (PIM) use in patients with CKD as high as 90% (Marin et al., 2020; Tesfaye et al., 2017). Furthermore, inappropriate polypharmacy is associated with adverse clinical outcomes, including adverse drug reactions (ADRs), poor medication⁣ adherence, increased hospitalisations, reduced quality of life, and increased mortality (Kimura et al., 2021). Such consequences can be minimised through targeted pharmaceutical interventions aimed at reducing inappropriate polypharmacy (Cooper et al., 2015).

Deprescribing interventions have demonstrated considerable success in reducing the harms associated with inappropriate polypharmacy in various populations, including older adults (Reeve et al., 2017). This highlights the need for deprescribing in populations where the consequences of inappropriate polypharmacy are well established, such as patients with CKD (Triantafylidis et al., 2018). Deprescribing is a structured, proactive process that involves discontinuation of inappropriate medications, reducing doses, or changing them with safer alternatives (Reeve et al., 2017). Despite the recognition of deprescribing as a core aspect of good prescribing practice, inappropriate polypharmacy still remains highly prevalent in the CKD population (Battistella et al., 2025). Several barriers hinder the effective implementation of deprescribing interventions in clinical practice. These include the lack of healthcare provider training, guidelines, and practical decision-support resources, especially in the CKD setting (Doherty et al., 2020).

In response, several initiatives have been undertaken to develop tools and resources to guide deprescribing in complex patient populations, including those with CKD (Zidan, ElGeed, et al., 2025). However, the limited availability of evidence-based, CKD-specific deprescribing algorithms that have been developed and validated for clinical practice presents a significant gap in optimising medication management in this population (Manski-Nankervis et al., 2021). We previously summarised and categorised existing evidence on deprescribing tools and guidelines for CKD through a scoping review (Zidan, ElGeed, et al., 2025). The identified tools included comprehensive deprescribing process guidance, protocols for comprehensive care models, drug-specific deprescribing algorithms (i.e. instructions and decision-support algorithms for structured deprescribing), and screening tools to identify targets for deprescribing.

Different methodological approaches have been used in developing deprescribing tools and guidelines, ranging from structured and rigorous processes to develop evidence-based clinical practice guidelines (e.g. systematic reviews followed by structured evidence synthesis, quality appraisal, testing, and validation) to more pragmatic approaches based on narrative literature reviews, with or without testing in clinical settings (Farrell et al., 2016; Zidan, ElGeed, et al., 2025). The identified drug-class-specific algorithms developed in Canada for the HD setting were validated and tested for implementation across several centres in Canada (Abbaticchio et al., 2025; Lefebvre et al., 2020). Recently, 50 high-risk medication toolkits were developed and validated to ensure safe prescribing for CKD patients in primary care (Halliday et al., 2025). However, these toolkits focus on the appropriate renal dose adjustments rather than on deprescribing (Halliday et al., 2025). Furthermore, the existing deprescribing tools do not adequately address the specific clinical needs of CKD patients outside the Canadian settings (Zidan, ElGeed, et al., 2025). In particular, commonly used general deprescribing guidelines are largely developed for broader or older adult populations and do not sufficiently account for CKD-related complexities such as altered pharmacokinetics and dialysis-related considerations that may warrant withdrawal or change in prescribed medications. Furthermore, the applicability and generalizability of the available CKD deprescribing tools are limited because they were mainly developed in Canada for patients on hemodialysis (HD) and have not yet been tested for utility in other clinical settings. Given the complexity of deprescribing interventions and the clear need for CKD-specific deprescribing resources, this study aimed to build on the available evidence to adapt, synthesise, and validate deprescribing algorithms that support clinicians in implementing deprescribing interventions in patients with KF.

## Methods

**Figure 1.**
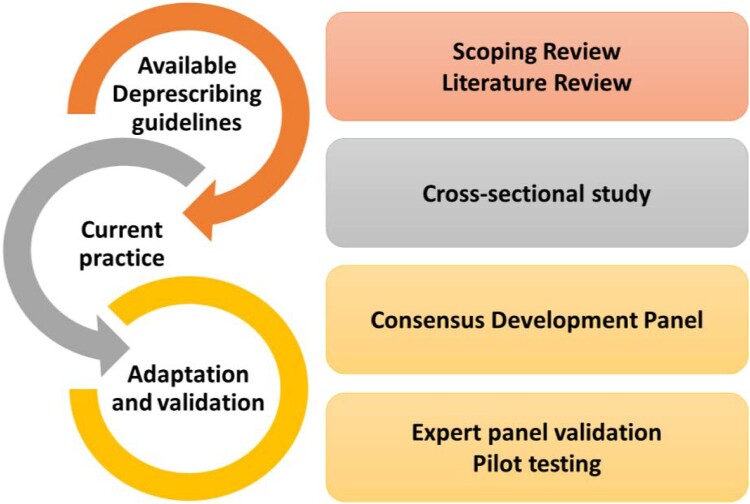
Overview of the processes used to develop and validate the KF-specific deprescribing algorithms.

This study was conducted in three phases. The first phase involved a scoping review of existing CKD deprescribing guides (Zidan, ElGeed, et al., 2025) and a review of deprescribing guidelines developed by global deprescribing networks to identify gaps and limitations in their applicability to KF populations. In addition, cross-sectional data from a random sample of prescriptions for patients receiving HD and pre-dialysis patients attending low-clearance clinics (LCC) [eGFR < 15 mL/min/1.73 m²] were analysed to identify the most commonly prescribed medications relevant to algorithm development. The second phase utilised a structured expert consensus meeting using a consensus development method (CDM) approach to develop comprehensive deprescribing algorithms for patients with KF. CDMs are appropriate for complex clinical contexts because they integrate available evidence with expert opinions through a systematic, structured and transparent process (Arakawa & Bader, 2022). Finally, the third phase involved validation of the developed algorithms through expert review and pilot testing in a pilot randomised controlled trial (pRCT) (Figure 1).

### Phase 1: evidence synthesis

#### Literature review

A literature review and findings from our previous work were used to identify deprescribing algorithms of high quality as assessed using the Appraisal of Guidelines for Research & Evaluation (AGREE II) tool (Brouwers et al., 2010) for CKD or the general older adult population. The details of the identified studies and critical appraisal results are published elsewhere (Zidan, ElGeed, et al., 2025). The information extracted from the retrieved articles included the developed tools, the medications classes targeted for deprescribing, and the development methods. Among the identified tools, we focused on decision-support algorithms because they provide structured and practical supporting tools for clinical decision-making. At this stage, a few medication classes were identified as targets for deprescribing. Those were the same as those focused on by the developed tools.

#### Current practice assessment

To understand prescribing patterns and identify the most commonly prescribed medications in KF, we reviewed a random sample of chronic prescriptions for 104 adults receiving HD and 50 adults receiving care at the low clearance clinic (LCC). These marked the target settings where our deprescribing algorithms are intended to be used. The number and therapeutic categories of the prescribed medications were summarised. The assessment did not include the medications prescribed for short periods (like antibiotics). These may include renally eliminated medications that warrant interventions. However, the focus of the intended algorithms is to deprescribe medications that are prescribed for long-term use. The primary focus was to identify the most frequently prescribed medication classes among the KF patients in the local setting.

### Phase 2: algorithm development

A panel of five experts in pharmacotherapy, medication safety, and clinical research was convened to evaluate the evidence and develop KF-specific deprescribing algorithms. The experts were invited based on their previous experience and recognition in the region for leading medication safety and deprescribing initiatives besides their track record of international collaboration. The development process comprised nine structured workshops during which the panel systematically reviewed, discussed, and iteratively refined draft algorithms. A predefined requirement of full consensus (100%) was applied for the inclusion of any recommendation. Prior to each expert workshop, two clinical pharmacists synthesised an evidence-based draft summarising deprescribing considerations for 2–3 drug classes. Evidence was drawn from existing CKD-specific deprescribing guidelines (George et al., 2021; Lefebvre et al., 2020; Gerardi et al., 2022) and updated using the most recent clinical practice guidelines from authoritative bodies, including Kidney Disease: Improving Global Outcomes (KDIGO), Kidney Disease Outcomes Quality Initiatives (KDOQI) (Levin et al., 2024), the American Heart Association (AHA) (Otto et al., 2025), and the American Diabetes Association (ADA) (American Diabetes Association Professional Practice Committee, 2025). Where appropriate, evidence from well-established deprescribing guidelines for the general population was also incorporated (Bruyere, 2025).

Each workshop followed a standardised format: (1) presentation of the evidence and identification of key gaps requiring expert judgement; (2) independent review and drafting of recommendations by panel members; (3) appraisal of the decision-support algorithm draft for content, flow, and logical structure; (4) discussion of the clinical suitability, feasibility, and practicality of each recommendation; (5) open voting of the recommendations, with 100% agreement required for inclusion; and (6) refinement and provisional approval of the updated algorithm component.

The resulting algorithms were designed to support clinicians in assessing medication appropriateness and guiding the deprescribing and monitoring process. A total of seven in-person and two online meetings were conducted, each lasting around 2–3 h.

### Phase 3: validation

#### Clinical expert validation

The developed algorithms were reviewed by an expert panel comprising two nephrology consultants, a nephrology fellow, and two clinical pharmacists with extensive clinical experience. The panel conducted several consensus meetings to evaluate the relevance, clarity, and practical usability of the algorithms. This expert review helped establish the content validity and face validity of the algorithms and informed refinements before their testing in the feasibility study. Proposed revisions were reached by full consensus among panel members, and the summarised feedback was communicated to the original development panel for incorporation into the final version. The clinical validation committee leader (nephrology consultant) had previously led the development of locally adopted MBD and anaemia management protocols, which have been successfully implemented in clinical practice (Hamad et al., 2021, 2024).

#### Pilot testing

The revised algorithms were evaluated in a deprescribing feasibility study (pilot randomised controlled trial – pRCT) involving adults with stage 5 KF. This pilot is part of a large deprescribing trial registered with ClinicalTrials.gov (identifier: NCT06324045) and has been published (Zidan, Hamad, et al., 2025). Participants (adults on maintenance HD or pre-HD, receiving care at ambulatory care centres) were randomised into either intervention or control groups. Participants in the intervention arm received a structured, pharmacist-led deprescribing intervention guided by the developed algorithms. Outcome measures of the p RCT were assessed at baseline, three months, and six months. The primary outcomes were feasibility and safety, while secondary outcomes included the number of potentially inappropriate medications (PIMs), pill burden, health service utilisation, medication adherence, treatment burden, and quality of life. Additionally, the frequency and appropriateness of algorithm use were documented. In the current study, we report the results of the algorithms development and use only. Issues identified during the pilot phase were reviewed, and necessary refinements were made. Following the completion of the validation and pilot stages, the development panel convened a final consensus meeting, using the same structured format, to finalise the algorithms. The refined algorithms will be used in a definitive interventional randomised controlled trial (RCT) of deprescribing in KF.

### Data synthesis

Descriptive analyses were conducted for the cross-sectional assessment and pilot RCT. Data were reported as frequencies and percentages for categorical variables and as means and standard deviations, as appropriate. Findings from the consensus development were reported narratively. Data management and descriptive analyses were performed using Microsoft Excel.

## Results

### Phase 1: evidence synthesis

#### Literature review

The literature review identified high-quality deprescribing algorithms developed by Lefebvre et al. (2020) and Gerardi et al. (2022), which were selected for adaptation during the consensus development meetings. In our previous critical appraisal, these algorithms achieved ≥70% of the overall AGREE II assessment. These tools provided structured decision-support algorithms with detailed guidance on standardising the deprescribing process, including recommendations for dose tapering, monitoring, and follow-up. They were developed and validated by Canadian pharmacists and nephrologists (Lefebvre et al., 2020) and showed positive effects in reducing the inappropriate use of medications (Gerardi et al., 2022). However, these algorithms focus on HD patients in the Canadian health system and were tested for clinical utility. In addition, we reviewed the most recent clinical practice guidelines relevant to the management of CKD (American Diabetes Association Professional Practice Committee, 2025; Levin et al., 2024; McEvoy et al., 2024; Otto et al., 2025; Van Gelder et al., 2024) as well as deprescribing guidelines developed by the Bruyère Research Institute (Bruyere, 2025) and Primary Health Tasmania (Primary Health Tasmania, 2022). This evidence base informed the initial framework used in the subsequent consensus development phase.

#### Current practice assessment

**Figure 2.**
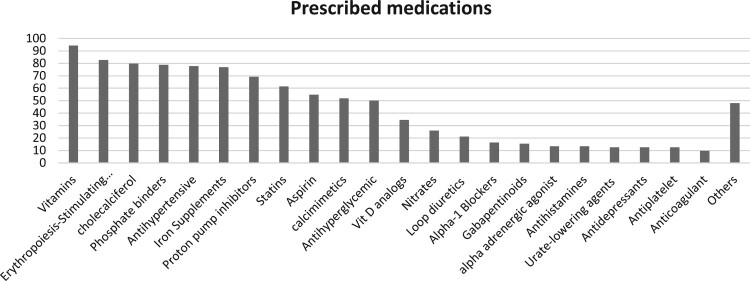
Prescribed medication classes in hemodialysis patients (%).

**Figure 3.**
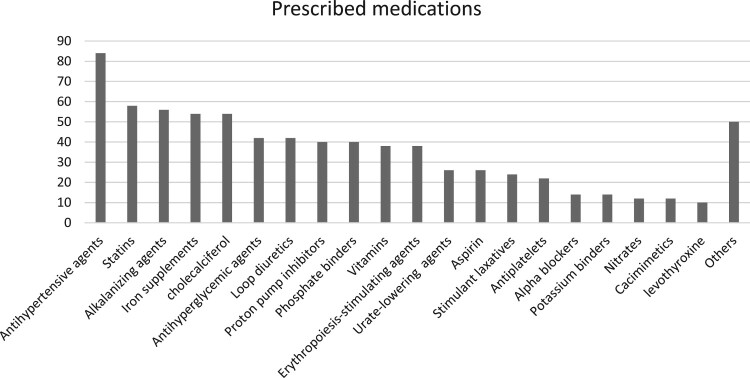
Prescribed medication classes in low-clearance clinic patients (%).

A total of 104 and 50 prescriptions for patients receiving HD and nephrology care at LCC, respectively, were included in the prescribing assessment. The mean (SD) age of the patients was 55.3 (13.7) years in the HD setting and 53.3 (10.5) years in the LCC setting. Males comprised 55.4% of the HD cohort and 69.5% of the LCC cohort. The mean (SD) number of comorbidities was 5.7 (2.4) among HD patients and 4.7 (1.3) among LCC patients. Polypharmacy was highly prevalent across both settings, with most patients receiving multiple chronic medications. In the HD cohort, the most frequently prescribed medication categories included vitamins (*n* = 98, 94%), anaemia and MBD therapies (erythropoiesis-stimulating agents: *n* = 86, 83%; cholecalciferol: *n* = 83, 80%; phosphate binders: *n* = 82, 79%), antihypertensive medications (*n* = 81, 78%), and proton pump inhibitors (*n* = 72, 69%). Similarly, in the LCC cohort, the most frequently prescribed drug classes were antihypertensive agents (*n* = 42, 84%), statins (*n* = 29, 58%), and alkalising agents (*n* = 28, 56%). Topical medications were not accounted for in this study. These prescribing patterns were discussed and appropriately considered during the algorithms' development phase (Figures 2 and 3).

In the HD cohort, medications prescribed in less than 10% of prescriptions included herbal products, laxatives, anticholinergic agents, nonsteroidal anti-inflammatory drugs (NSAIDs), benzodiazepines, corticosteroids, antacids, antipsychotics, aminosalicylates, phosphodiesterase-5 (PDE-5) inhibitors, vasodilators, levothyroxine, analgesics, anticonvulsants, venoactive bioflavonoids, ezetimibe, electrolyte replacement therapies, potassium binders, leukotriene receptor antagonists, antivirals, betahistine, immunosuppressants, antiarrhythmics, dopamine agonists, and sedatives. Similarly, for the LCC cohort, medications prescribed in fewer than 10% of prescriptions included herbal products, anticholinergic agents, corticosteroids, antiflatulents, quinine, PDE-5 inhibitors, analgesics, anticonvulsants, cholesterol absorption inhibitors, electrolyte replacement therapies, leukotriene receptor antagonists, histamine analogues, immunosuppressants, antiarrhythmics, vitamin D analogues, and sedatives.

### Phase 2: algorithm development

Nineteen evidence-based deprescribing algorithms were developed through expert consensus meetings conducted between December 2023 and January 2024. These included one general (i.e. generic) deprescribing algorithm applicable to any medication and 18 drug class-specific algorithms tailored for patients with KF (Table 1). Although not all of the selected drug classes were common in the cross-sectional prescribing analysis, the expert panel agreed to include them to ensure broader applicability in KF population and to provide guidance for clinical cases in which such medications may still be prescribed. The algorithms were designed to support clinicians in systematically evaluating medication appropriateness and in implementing a structured, stepwise deprescribing process with appropriate monitoring and follow-up. Subsequent clinical expert validation and pilot testing led to further refinements, confirming the algorithms’ usability and clinical utility. All algorithms address two key decision points: whether a medication should be deprescribed, and how the deprescribing process should be conducted.

**Table 1.** Summary of the developed deprescribing algorithms and rationale for inclusion.

Type	Drug class	Reason for inclusion	Frequency of prescribing*	Key references
Generic	–	Essential for initial medication review and the deprescribing process.	–	- Medication Appropriateness Index (MAI) (Hanlon & Schmader, 2013). - Generic Deprescribing Framework (Reeve et al., 2017; Scott et al., 2015)
Drug-class specific	Alpha-1 blockers	Commonly prescribed in CKD; added for comprehensiveness	Less common	- Lefebvre et al. Deprescribing Algorithms (Lefebvre et al., 2020) - Gerardi et al. Deprescribing Algorithms (Gerardi et al., 2022)
	Anticholinergics	High-risk in CKD and older adults	Rare	- A Guide to Deprescribing Anticoagulants (Primary Health Tasmania)
	Anticoagulants	High prevalence of AF and vascular disease in CKD – added for comprehensiveness	Rare	- A Guide to Deprescribing Anticoagulants. (Primary Health Tasmania) - European Society of Cardiology Atrial Fibrillation Guidelines (2024) (Van Gelder et al., 2024)
	Antihyperglycemics	Relevant to CKD population	Common to very common	- Gerardi et al. Deprescribing Algorithms - A Guide to deprescribing Antihyperglycemics. (Primary Health Tasmania)
	Antihypertensives	Core CKD therapy	Very common	- A Guide to deprescribing antihypertensives.(Primary Health Tasmania) - European Society of Cardiology Hypertension Guidelines (2024) (McEvoy et al., 2024)
	Antiplatelets	Common cardiovascular comorbidity	Less common	- Gerardi et al. Deprescribing Algorithms - A Guide to deprescribing antiplatelets (Primary Health Tasmania)
	Antipsychotics	Potentially inappropriate; high-risk	Rare	- A guide to deprescribing antipsychotics (Primary Health Tasmania) - Lefebvre et al. Deprescribing Algorithms
	Aspirin	Widely prescribed CVD therapy	Less common to very common	- Gerardi et al. Deprescribing Algorithms
	Benzodiazepines	High-risk PIMs; included to support deprescribing even if infrequent	Rare	- Lefebvre et al. Deprescribing Algorithms Gerardi et al. Deprescribing Algorithms - KDIGO clinical practice guideline - A Guide to deprescribing benzodiazepines (Primary Health Tasmania)
	Gabapentinoids	Frequently used for neuropathy	Rare to less common	- Lefebvre et al. deprescribing algorithms - A Guide to deprescribing gabapentinoids (Primary Health Tasmania)
	Long-acting nitrates	CVD comorbidities	Less common	- A Guide to deprescribing long-acting nitrates (Primary Health Tasmania)
	Loop diuretics	Key therapy in CKD management	Less common to common	- Lefebvre et al. deprescribing algorithms - Gerardi et al. deprescribing algorithms - KDIGO clinical practice guideline
	Non-steroidal ani-inflammatory drugs (NSAIDs)	Contraindicated or harmful in CKD	Rare	- A Guide to deprescribing non-steroidal anti-Inflammatory drugs (NSAIDs)
	Prokinetics	GI symptoms frequent	Rare	- Lefebvre et al. deprescribing algorithms
	Proton pump inhibitors (PPIs)	Common in CKD + known PIMs	Common to very common	- Bruyer’s PPI deprescribing guideline (Farrell et al., 2017) - Lefebvre et al. deprescribing algorithms - Gerardi et al. deprescribing algorithms - A guide to deprescribing proton pump inhibitors (PPIs) (Primary Health Tasmania)
	Quinine	Included for broad coverage	Rare	Lefebvre et al. deprescribing algorithms
	Statins	Widespread use	Very common	- Lefebvre et al. deprescribing algorithms - Gerardi et al. deprescribing algorithms - KDIGO Clinical Practice Guidelines - A Guide to deprescribing statins (Primary Health Tasmania)
	Urate-lowering agents	Used in CKD gout	Less common	- Lefebvre et al. deprescribing algorithms - Gerardi et al. deprescribing algorithms - KDIGO Clinical Practice Guidelines - A Guide to deprescribing urate-lowering agents (Primary Health Tasmania).

*Based on the cross-sectional study: [Rare: <10%, less common: (10-29%), common: (30-49%) &, and very common: ≥50% of the prescriptions of both settings].

#### Generic deprescribing algorithm

**Figure 4.**
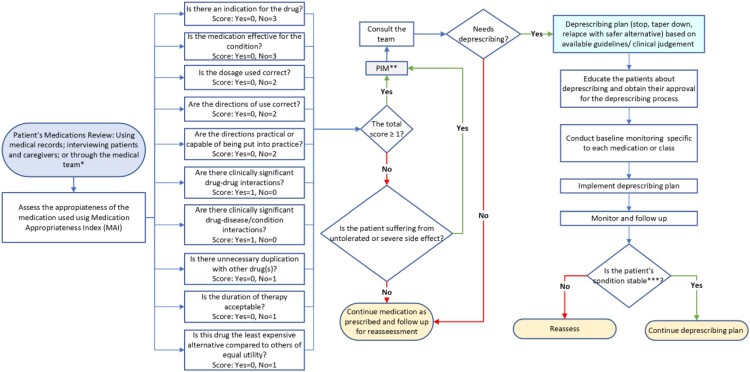
Generic deprescribing algorithm *Please confirm patient adherence and ensure that he/she is taking the medication as prescribed before using the algorithm. ** Potentially Inappropriate Medication ***Stable patient condition: The individual's health is consistently maintained, with no significant deterioration or rapid fluctuations observed over time. The patient's condition is relatively stable, with vital signs, symptoms, and overall clinical status within an acceptable range, given their underlying health condition. Additionally, the patient is not experiencing any withdrawal or adverse events.

The generic deprescribing algorithm guides clinicians through a structured medication appropriateness review (screening phase) and outlines the actions required when deprescribing is indicated. Key items from the validated Medication Appropriateness Index (MAI) (Hanlon & Schmader, 2013) were incorporated to support systematic assessment during medication review and reconciliation. Moreover, a protocol for conducting the deprescribing process within a multidisciplinary care approach was integrated. Notably, deprescribing was considered a therapeutic trial in all cases, requiring planned monitoring (Figure 4).

#### Drug class-specific algorithms

Drug class-specific algorithms were developed using the same structured decision-making approach to address the two core questions within each therapeutic class (whether to deprescribe and how to deprescribe). The following medication classes were selected during the initial consensus phase based on prescribing patterns in KF: alpha-1 blockers, anticholinergic agents, anticoagulants, antidepressants, antihyperglycemic agents, antihypertensive agents, antiplatelets, antipsychotics, aspirin, benzodiazepines, gabapentinoids, long-acting nitrates, loop diuretics, NSAIDs, prokinetics, PPIs, quinine, statins, and urate-lowering agents (Supplementary Material).

### Phase 3: validation

#### Expert validation

An expert panel (two nephrology consultants, one nephrology fellow, one cardiology clinical pharmacist, and one nephrology clinical pharmacist) independently reviewed each developed deprescribing algorithm. The panel subsequently convened for consensus meetings to discuss all identified issues. The primary objective of those meetings was to evaluate the usability, practicality, and applicability of the algorithms within routine clinical practice. Besides, the clinical pharmacist closely examined the content of the algorithms against clinical practice guidelines. Overall, the panel considered the algorithms highly valuable and recommended their adoption at the institutional level. They agreed that clinical pharmacists are the most appropriate healthcare professionals to lead the application of the algorithms, with all deprescribing decisions communicated and coordinated through the multidisciplinary team (MDT). They indicated that the use would reduce the clinician’s time burden while conducting comprehensive medication review and proposing deprescribing plans. The experts provided detailed recommendations regarding clinical content, formatting, and real-world usability of the algorithms, drawing on their experiences in managing patients with KF. All consensus-based recommendations were communicated to the development team and incorporated into the subsequent revisions of the algorithms.

However, the panel noted that not all the algorithms were directly applicable within their current clinical context. Specifically, certain medication classes, such as anxiolytics (benzodiazepines and Z drugs) and antipsychotics, fall outside the nephrology division's prescribing or deprescribing authority, and these agents are not routinely modified in their practice setting. Furthermore, not all recommendations were practical for utility in the clinical setting. Refinements suggested to the algorithms are summarised in Table 2.

**Table 2.** Summary of expert feedback and corresponding revisions to the deprescribing algorithms.

Algorithm	Issue identified	Implemented revisions
All algorithms	The ‘unclear indication’ definition contained excessive wording.	Wording was reduced and simplified to improve usability.
Statins	Recommendation to reassess therapy every 3 months was considered impractical.	Reassessment frequency modified to ‘as clinically indicated.’
	Ezetimibe pathway required clarification.	Algorithm revised to reflect that ezetimibe may also be used in primary prevention.
	Insufficient guidance for non-HD patients.	Additional stratification added based on age, diabetes status, and ASCVD risk.
	Hepatic dose adjustment considerations were not specified.	Hepatic dose adjustment information added.
Urate-lowering agents	Monthly reassessment after discontinuation was considered impractical.	Follow-up modified to one assessment at 1 month after discontinuation.
	Colchicine contraindicated in HD; prophylaxis duration for anti-inflammatory therapy required clarification.	Algorithm revised to indicate colchicine contraindication in HD and updated management for gout attacks in patients with contraindications.
	Monitoring of serum uric acid levels was not specified for non-HD patients.	Baseline uric acid monitoring added for non-HD patients.
	Clarification required regarding continuation of allopurinol in HD vs. non-HD patients.	Algorithm updated to distinguish between HD and non-HD pathways.
Aspirin	Clarification required regarding aspirin indication criteria.	Algorithm revised to reflect updated indication guidance.
	Primary prevention: Unclear management when prescribed for patients <70 years but with low/intermediate ASCVD risk.	Algorithm revised to clarify risk-based decision-making for primary prevention.
Proton pump inhibitors (PPIs)	Final decision step regarding symptom improvement required clarification.	Wording revised based on expert recommendation.
	Consideration of dual antiplatelet therapy (DAPT) as an indication for PPIs was debated.	Clarified that DAPT alone is not a routine indication unless additional risk factors are present (e.g. steroid use, anticoagulants, ulcer history).
Long acting nitrates	Heart failure (HF) indication was missing.	HF indication added to the nitrate algorithm.
	Tapering strategy and dose criteria required clarification.	Dose-reduction percentages and revised tapering criteria added.
Loop diuretics	Additional clarification required for appropriate use in CKD/HD patients.	Explanatory note added to loop diuretics algorithm.
Antiplatelets	Algorithm considered redundant because recommendations align directly with existing clinical guidelines (e.g. dual antiplatelet therapy).	Decision made not to further expand this algorithm, as guidance is already established in clinical guidelines.
Antihyperglycemic agents	Linagliptin continuation unclear in patients receiving intensive insulin therapy.	Recommendation added that linagliptin may be discontinued if the patient is receiving two insulin regimens.
	The algorithm suggested deprescribing when hypoglycemia occurs; however, some patients experienced hypoglycemia while still having poorly controlled glucose levels.	The algorithm was not fully implemented during the feasibility phase. Deprescribing decisions were instead guided by the multidisciplinary team (MDT) clinical judgment, with referral to endocrinology or diabetes educators when necessary.
	It also recommended monitoring every two weeks when glucose is controlled, which was not practical.	
	In addition, GLP-1 receptor agonists were suggested as alternatives despite limited accessibility due to high cost and restricted coverage.	
	Monitoring focused on hypoglycemia without addressing hyperglycemia, which was more clinically relevant.	

#### Pilot testing

The revised algorithms were subsequently used in a feasibility pilot randomised controlled trial (pRCT) conducted between February 2024 (start of patient recruitment) and July 2025 (end of follow-up). The primary aim of the pilot was to determine the feasibility and safety of delivering a structured deprescribing intervention, using the developed algorithms as guidance for clinical pharmacists and the MDT. The pRCT was conducted in two clinical settings: HD units and LCC.

The findings of the pRCT regarding the feasibility of recruitment and safety of the intervention will be reported separately. Briefly, the intervention delivery was feasible and safe, supporting progression to a definitive RCT. This was demonstrated by recruiting 72% of the screened patients, delivering all the intervention components with no serious adverse events, and having no more than 5.6% withdrawals.

Observations regarding the algorithms’ use during the pRCT indicate that the deprescribing algorithms for KF were generally acceptable, easy to use, and operationally practical. Within the research team, the clinical pharmacist led the medication review process and drafted deprescribing plans in collaboration with the MDT. Table 3 represents the frequency and nature of algorithm use during the pilot phase. During the pilot stage, a total of 74 and 11 deprescribing plans were proposed in the HD and LCC settings, respectively. Of them, 41 plans (55.4%) in the HD setting and 5 plans (45.5%) in the LCC setting were formulated using the drug-class specific algorithms. The generic deprescribing algorithm was applied in all cases.

**Table 3.** The use of the developed deprescribing algorithms in the pilot feasibility study in hemodialysis and low-clearance clinic patients.

Algorithm used	Medications	The purpose of using	Frequency in HD	Frequency in LCC	Comments
Generic deprescribing algorithm Drug class specific	Antihypertensive agents	Decide to deprescribe or not	18	0	The algorithm is of a generic nature – each antihypertensive class needed a different clinical assessment.
	PPIs	- Decide to deprescribe or not - Tapering schedule	15	1	Appropriate with minor edits suggested
	Statins	- Decide to deprescribe or not	8	1	Appropriate with minor edits suggested
	Antiplatelets and aspirin	- decide to deprescribe or not	7	1	Appropriate – compatible with clinical practice guidelines
	Antihyperglycemic medications	- Decide to deprescribe or not	2	1	Not fully used
	Loop diuretics	- Decide to deprescribe or not - Monitoring	5	0	Appropriate with minor edits suggested
	Alpha-1 blocker	- Decide to deprescribe or not	3	0	Appropriate with minor edits suggested
	Urate-lowering agents	- Decide to deprescribe or not - Monitoring	1	1	Appropriate with minor edits suggested
Generic deprescribing algorithm	Vitamins and supplements	- Decide to deprescribe or not	3	1	Appropriate
	Antihypertriglyceridemic	- Decide to deprescribe or not - How to deprescribe	1	0	
	Hepatic medications	- Decide to deprescribe or not - How to deprescribe	1	0	
	Obesity medications	- Decide to deprescribe or not - How to deprescribe	2	0	
	Electrolyte supplements	- Decide to deprescribe or not - How to deprescribe	0	1	
	Thiazide diuretics	- Decide to deprescribe or not - How to deprescribe	0	1	
	Betahistine	- Decide to deprescribe or not - How to deprescribe	1	0	
Generic deprescribing algorithm HMC local management protocol	Mineral bone disease medications*	- Decide to deprescribe or not - How to deprescribe	7	2	Appropriate with minor edits suggested
	Anaemia medications**	- Decide to deprescribe or not - How to deprescribe	0	1	
	Total		74	11	

*Vitamin D analogues, calcimimetics, calcium supplements, phosphate binders, and vitamin D3 supplements. **Erythropoietin-stimulating agents and iron supplements.

PPI, proton pump inhibitors.

Notably, while the algorithms supported and streamlined clinical decision-making, professional clinical judgment remained essential. Their primary contribution was in reducing clinicians’ time burden and providing a structured, standardised framework to support deprescribing decisions. In addition, the study setting employed established internal protocols for MBD, anaemia management, and other therapeutic agents (e.g. potassium binders and immunosuppressants). These protocols were used alongside the deprescribing algorithms during the pRCT and provided practical context-specific deprescribing guidance tailored to local clinical practice.

## Discussion

Research on deprescribing has traditionally focused on older adult populations (Reeve et al., 2015). Recently, however, attention has shifted to other patient populations (Dixon et al., 2022; Gupta & Cahill, 2016; Jahan et al., 2019), including individuals with CKD. These later groups are often underrepresented in clinical trials, which limits the evidence on medication safety and efficacy for this group (Colombijn et al., 2023). Additionally, deprescribing research in CKD settings remains in its early stages. This study aimed to synthesise and adapt the available evidence into practical decision-support algorithms to guide clinicians in delivering deprescribing interventions for patients with KF. Drug-class-specific algorithms were selected as the primary form of decision support, given their demonstrated practicality and applicability in busy clinical environments (Jia et al., 2016). The development process involved a comprehensive review of the current literature, building on existing high-quality algorithms, and integrating relevant deprescribing recommendations derived from clinical guidelines developed for the general population. Additionally, we conducted a cross-sectional study to characterise current prescribing patterns and prioritise medication classes most relevant to KF care.

Given the extensive number of medications prescribed to patients with KF, it was not feasible to establish evidence-based guidance for every medication class. Consequently, we developed a generic deprescribing algorithm integrating screening for medication appropriateness and a structured, stepwise approach to deprescribing and monitoring, irrespective of medication type. The MAI was incorporated as an implicit tool due to its high sensitivity and reliability in identifying inappropriate prescribing across various populations (Hanlon & Schmader, 2013, 2022).

To guide the development of the algorithms, we used a consensus development methodology. Such methods are well established for addressing complex clinical decisions (Arakawa & Bader, 2022; Nazar et al., 2018). The nature of the evidence, combined with the need for contextual adaptation, necessitated expert input and formal consensus-driven procedures. We further conducted a two-step validation of the developed algorithms. While there is no gold standard method for decision-support algorithms validation (Lefebvre et al., 2020), clinical expert validation and pilot testing were deemed appropriate in this study. Expert review offered insight into practical implementation and application, content relevance, and potential end-users (Nagavci et al., 2025), while pilot testing in real clinical settings provided an authentic assessment of feasibility, usability, and potential limitations requiring refinements (Hoddinott, 2015).

Our findings align with and build upon prior deprescribing algorithm development and validation studies. Lefebvre et al. developed nine deprescribing algorithms through a systematic process that involved literature review and content and face validation using the Lynn method with 45 nephrology clinicians (Lefebvre et al., 2020). We adapted these algorithms with permission and expanded them by incorporating new content relevant to the KF context. Importantly, unlike the study by Lefebvre *et al*., our adapted algorithms were further evaluated through pilot testing in real-world clinical settings.

Similarly, Gerardi et al. adapted and developed deprescribing algorithms using evidence from the literature and applied them within a quasi-experimental intervention, demonstrating their feasibility in clinical practice (Gerardi et al., 2022). In contrast, our work employed a structured consensus methodology throughout adaptation, development, and validation. George *et al*. developed a comprehensive deprescribing tool for HD patients through a systematic review of current HD treatment recommendations and piloted it in 25 patients before implementing it in 150 patients in a pre- post study (George et al., 2021). Together, these studies support the practicality of algorithm-based deprescribing approaches, while our work adds further evidence by integrating expert validation and pilot randomised testing in KF populations. While our study shared the focus on comprehensive deprescribing and real-world testing, our approach was methodologically structured and rigorous.

In another important example, Halliday et al. recently developed and validated over 50 toolkits and algorithms to guide community pharmacists caring for patients with CKD regarding the use of renally eliminated medications (Halliday et al., 2025). Our focus is different in that we concentrated on the process of assessing and deprescribing chronic medications among patients with KF, rather than on the general CKD population and renally eliminated medications.

To our knowledge, we provided a significant contribution to the deprescribing research in the CKD setting. Although our work builds upon prior studies, it extends them by emphasising practical usability, structured adaptation, and validation through a rigorous pilot RCT. However, the study has several limitations. First, we relied on adapting the available deprescribing tools for CKD, which were themselves limited by evidence derived from the general population. Our study, therefore, inherited these same limitations. Second, while consensus methods are valuable, they inherently introduce risks of subjectivity. To mitigate these, we conducted multiple consensus rounds, applied a stringent requirement for anonymous agreement, and complemented expert validation with pilot testing. Third, we did not incorporate guidance on renally adjusted medications. While this is crucial for CKD care and medication safety, our focus in this study was to find guidance on reducing the burden of inappropriate medications prescribed for a long period. Other works may also become available and might be beneficial for refining our algorithms. Future updates and refinements will include such evidence. Fourth, in our cross-sectional assessment, we attempted to explore the prescribing trends; however, the inclusion of 104 prescriptions from HD and 50 from LCC may not be completely informative for algorithm development and may limit the generalizability of the results. However, the pilot testing provided better evidence. Further research is required to test their usability, suitability, and validity in other settings. Lastly, we did not include patient representatives in the development process. Although patient involvement is an important component of tool development (Bridges et al., 2018; Lefebvre et al., 2020), logistical constraints made this practically challenging in our setting. Future research should ensure patient representation and explore implementation strategies that influence policy adoption. Expert reviewers highlighted that clinical pharmacists may be the ideal end-users of these algorithms, given their central role in medication review and multidisciplinary decision-making. This warrants further exploration in future implementation studies.

## Impact on practice

This study contributes meaningfully to global efforts to advance evidence-based deprescribing in patients with CKD. Implementation of deprescribing interventions in this high-risk population requires a structured process supported by decision-support algorithms. Our attempt to develop and validate structured and clinically relevant deprescribing algorithms provided a practical resource for clinicians seeking to implement proactive deprescribing. While the algorithms would benefit from further validation studies, clinicians should always seek structured tools for such complex interventions like deprescribing.

## Conclusion

This study provided evidence on the benefit of using deprescribing algorithms tailored to patients with KF, offering a structured framework to support clinicians in identifying and safely deprescribing potentially inappropriate medications.

## Supplementary Material

Supplemental Material
