# Supplementary material for: Development and validation of deprescribing algorithms for kidney failure using consensus development methodology
Source: J Pharm Policy Pract. 2026 May 20;19(1):2666250. doi: 10.1080/20523211.2026.2666250 (PMC13192121; doi:10.1080/20523211.2026.2666250)
Supplement: Supplemental Material [file JPPP_A_2666250_SM2658.docx]

**Deprescribing Algorithms for Patients with Chronic Kidney Disease**

Amani Zidan^a^, Ph.D. candidate, MSc., Kheloud Awad^b^, PharmD, Safeya Habib^c^, PharmD, Noor Alsalemi^c^, PharmD, Ph.D., Hager El-Geed^c^, PharmD, Abdullah Hamad^d^, MD, Hassan Al-Malki^d^, MD, Mohamad Alkadi^d^, MD, Mohamed Elesnawi^d^, MD, Fatima Babiker^d^, MD, Rania Ibrahim^d^, MSc, Ahmed Awaisu*^c^, Ph.D.

^a^ QU-Health, Qatar University, Doha, Qatar

^b^ Pharmacy Department, Heart Hospital, Hamad Medical Corporation, Doha, Qatar

^c^ College of Pharmacy, QU-Health, Qatar University, Doha, Qatar

^d^ Division of Nephrology, Department of Medicine, Hamad Medical Corporation, Doha, Qatar.

# Table of Contents

[Table of Contents 2](#_Toc187490917)

[Introduction: 4](#_Toc187490918)

[Generic deprescribing framework: 5](#_Toc187490919)

[Drug-class specific algorithms 8](#_Toc187490920)

[1. Quinine 8](#_Toc187490921)

[2. Loop diuretics 10](#_Toc187490922)

[3. Alpha-1 blocker 12](#_Toc187490923)

[4. Statins 14](#_Toc187490924)

[5. Proton Pump Inhibitors (PPIs) 16](#_Toc187490925)

[6. Benzodiazepine and Z-drugs 18](#_Toc187490926)

[7. Urate-lowering agents 20](#_Toc187490927)

[8. Prokinetic agents 22](#_Toc187490928)

[9. Gabapentinoids 24](#_Toc187490929)

[10. Oral Anti-hyperglycemic 26](#_Toc187490930)

[11. Antihypertensive agents 28](#_Toc187490931)

[12. Anticoagulants 30](#_Toc187490932)

[13. Aspirin 32](#_Toc187490933)

[14. Antiplatelets 33](#_Toc187490934)

[15. Long-acting Nitrates 34](#_Toc187490935)

[16. NSAIDs 35](#_Toc187490936)

[17. Anticholinergic agents 36](#_Toc187490937)

[18. Antipsychotic agents 37](#_Toc187490938)

[Resources: 38](#_Toc187490939)

# Introduction:

Deprescribing is a structured approach to managing the consequences of inappropriate polypharmacy [1, 2]. Patients with chronic kidney disease (CKD) are particularly vulnerable to inappropriate polypharmacy due to the numerous CKD-related complications and comorbidities they experience [2]. However, clinical practice guidelines provide limited evidence on discontinuing medications as diseases progress and change over time [3].

This document serves as a guide for healthcare professionals to implement deprescribing interventions for patients with CKD. It is based on the best available evidence from deprescribing research in CKD and the general population, as well as evidence-based resources on prescribing, deprescribing, and CKD management guidelines. The available deprescribing algorithms in CKD were adapted for this guide after necessary permissions granted by their developers [4].

Deprescribing should be approached as a therapeutic trial, with regular monitoring and follow-up over specific periods to evaluate patient outcomes [2]. This guide emphasizes a multidisciplinary approach, highlighting the importance of involving a multidisciplinary team (MDT) to effectively implement deprescribing interventions [5]. In addition, patient education has been considered a core step in this guide as the evidence showed its impact on the success of deprescribing [6].

The guide includes two sections:

1. A generic deprescribing algorithm for reviewing medications and identifying potentially inappropriate medications.
2. Eighteen drug-specific deprescribing algorithms outlining step-by-step approaches to discontinue specific medications safely. Each algorithm contains the following steps:

1) Medication Review, 2) Medication Appropriateness, 3) MDT Consultation, 4) Deprescribing Plan Development, 5) Patient Education, 6) Baseline Monitoring, and 7) Safety and Efficacy Monitoring and Evaluation. The details of the steps may differ based on the medications and the patient’s needs.

# Generic deprescribing framework:

**
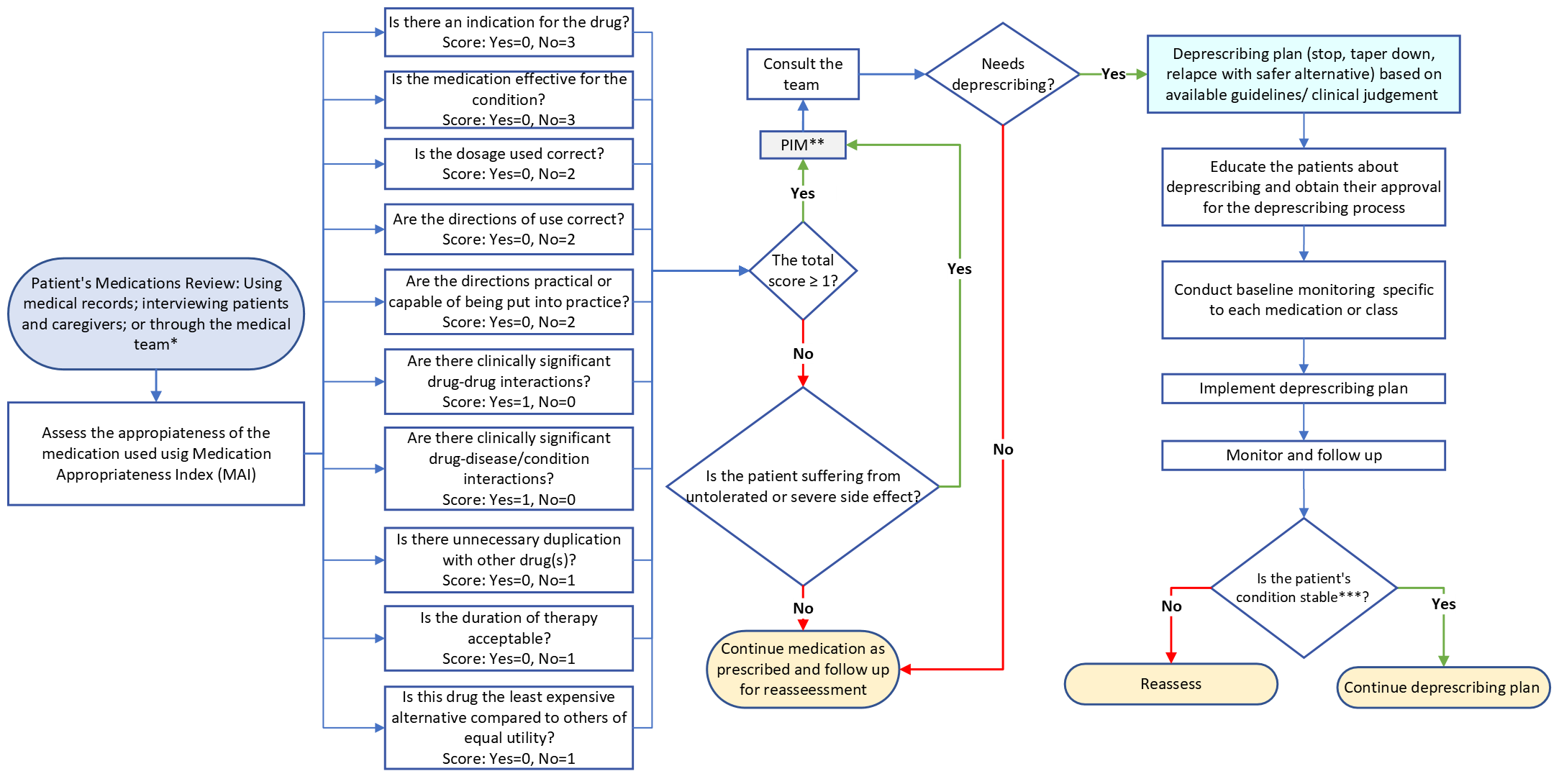
**

**Consulting the team includes the specialty teams (e.g. cardiology, endocrinology, and vascular)**

*****Please confirm patient adherence and ensure that he/she is taking the medication as prescribed before using the algorithm.
**^**^** Potentially Inappropriate Medication

**^***^**Stable patient condition: The individual's health is consistently maintained, with no significant deterioration or rapid fluctuations observed over time. The patient's condition is relatively stable, with vital signs, symptoms, and overall clinical status within an acceptable range, given their underlying health condition. Additionally, the patient is not experiencing any withdrawal or adverse events.

1. **Patient’s medication review:** Perform a comprehensive medical and medication review to assess the patient’s medication using electronic medical records, patient interviews, and collaboration with the MDT.
2. **Medication appropriateness assessment:** Assess and evaluate the appropriateness of the medications using Medication Appropriateness Index (MAI) calculator ( <https://l1nq.com/ElJ2d> ) [7, 8]. MAI estimates the appropriateness of medication by evaluating different aspects of medication use. These include: indication, effectiveness, dosage, directions, drug-drug interactions, and adverse effects. Untolerated and severe adverse drug reaction was taken into consideration since these are not covered by the calculator.
3. **Assess the need for deprescribing:**

- MAI score ≥ 3; the medication is considered potentially inappropriate medication (PIM); consider deprescribing.
- MAI score < 3 with any identified factor for deprescribing (side effect, dose reduction, education,...); the medication is considered potentially inappropriate medication (PIM); consider deprescribing
- MAI score < 3 without any identified factor for deprescribing; continue the medication as prescribed.

1. **Addressing potentially inappropriate medications:**

- Discuss the findings with the MDT.
- Consider conducting baseline tests if needed.

1. **Patient Education**: Educate the patients about deprescribing and obtain their approval for the deprescribing process.
2. **Deprescribing plan development**:

- Develop a deprescribing plan based on the guidelines provided below and the clinical judgment of the MDT.
- Prioritize the targets for deprescribing based on the identified clinical indicators.
- Conduct baseline monitoring if needed.
- Deprescribing includes stopping the medication, tapering down the dosage, or switching to a safer alternative.
- Educate the patient on the proper use of medications.

1. **Monitoring and evaluation:**

- Monitor and follow-up the patient closely during and after implementing the deprescribing plan and evaluate the patient’s response.
- Based on the patient’s response, decide with the MDT whether to continue with the deprescribing plan or re-initiate the medication.

# Drug-class specific algorithms

##
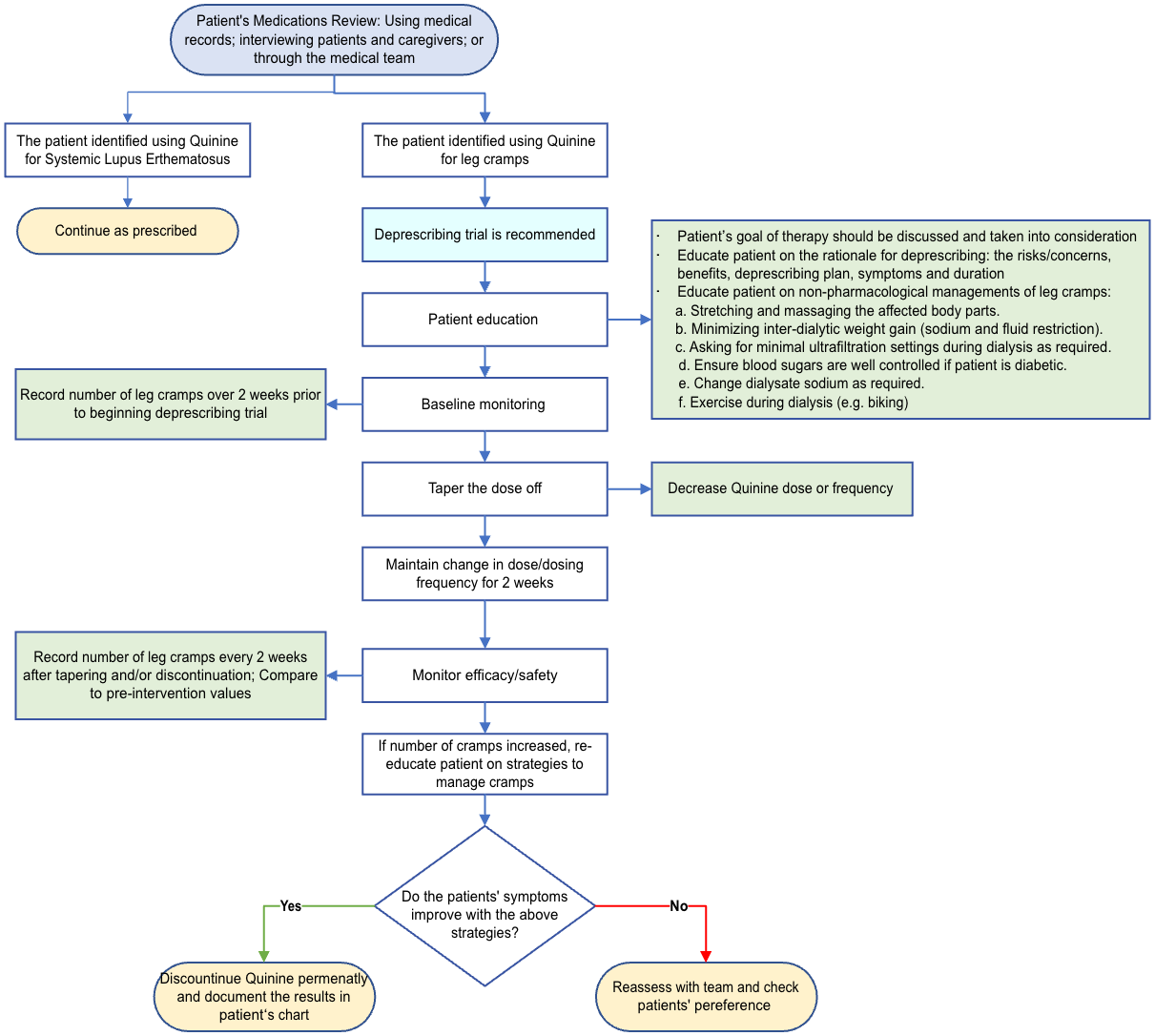
Quinine

**References**:

Lefebvre, M.J., et al., *Development and validation of nine deprescribing algorithms for patients on hemodialysis to decrease polypharmacy.* Canadian Journal of Kidney Health and Disease, 2020. **7**: p. 2054358120968674

## Loop diuretics


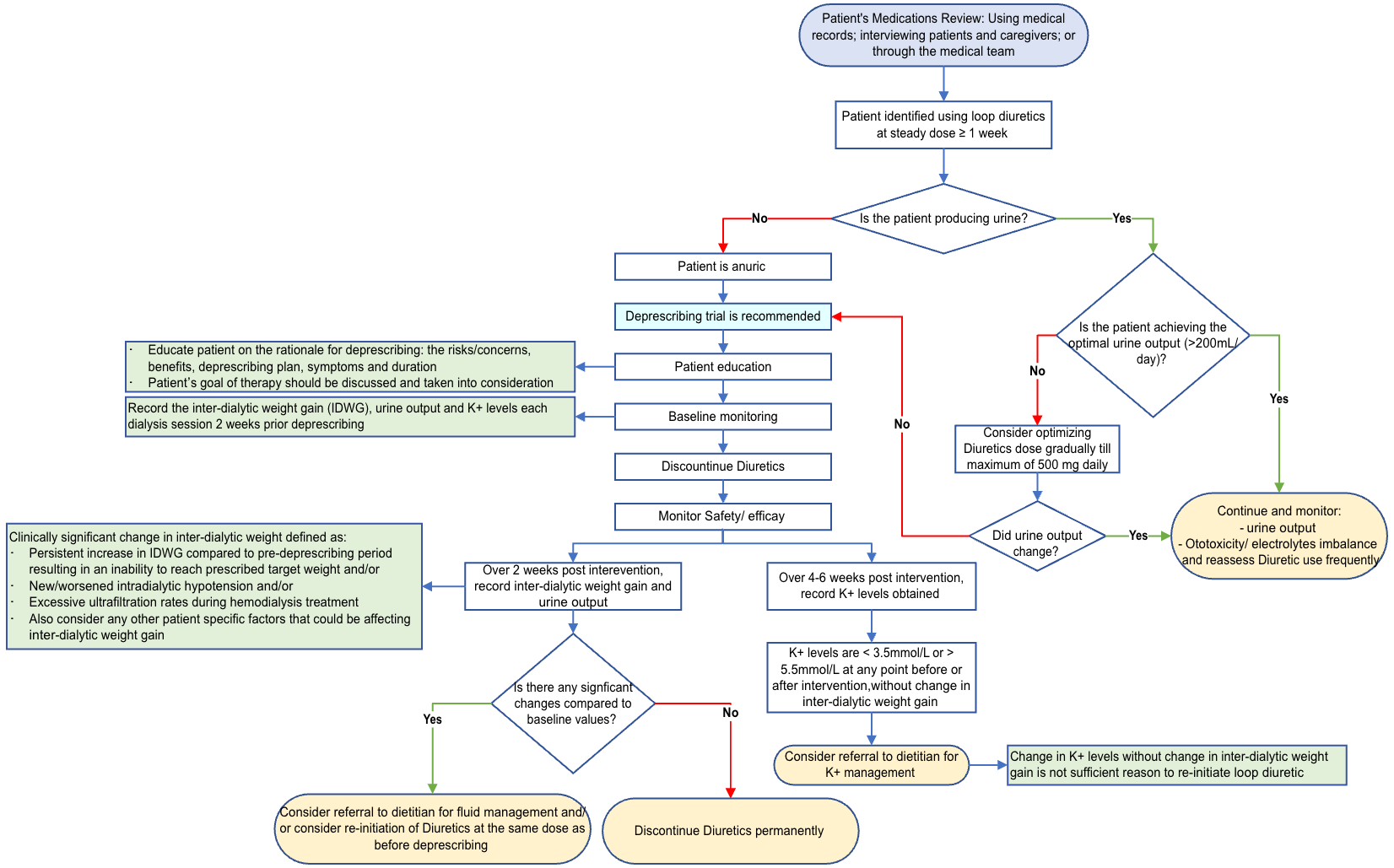


Note: the algorithm applies to HD patients, as the need for diuretics in non-HD CKD patients is assessed clinically.

**References:**

- Lefebvre, M.J., et al., *Development and validation of nine deprescribing algorithms for patients on hemodialysis to decrease polypharmacy.* Canadian Journal of Kidney Health and Disease, 2020. **7**: p. 2054358120968674.
- Gerardi, S., et al., *Implementation of targeted deprescribing of potentially inappropriate medications in patients on hemodialysis.* American Journal of Health-System Pharmacy, 2022. **79**(Supplement_4): p. S128-S135.
- Cheung, A.K., et al., *KDIGO 2021 clinical practice guideline for the management of blood pressure in chronic kidney disease.* Kidney International, 2021. **99**(3): p. S1-S87.
- <https://online-lexi-com.eu1.proxy.openathens.net/lco/action/doc/retrieve/docid/hamad_f/3022845?cesid=3F8Is4sMZVE&searchUrl=%2Flco%2Faction%2Fsearch%3Fq%3Dfurosemide%26t%3Dname%26acs%3Dtrue%26acq%3Dfurose>

## Alpha-1 blocker


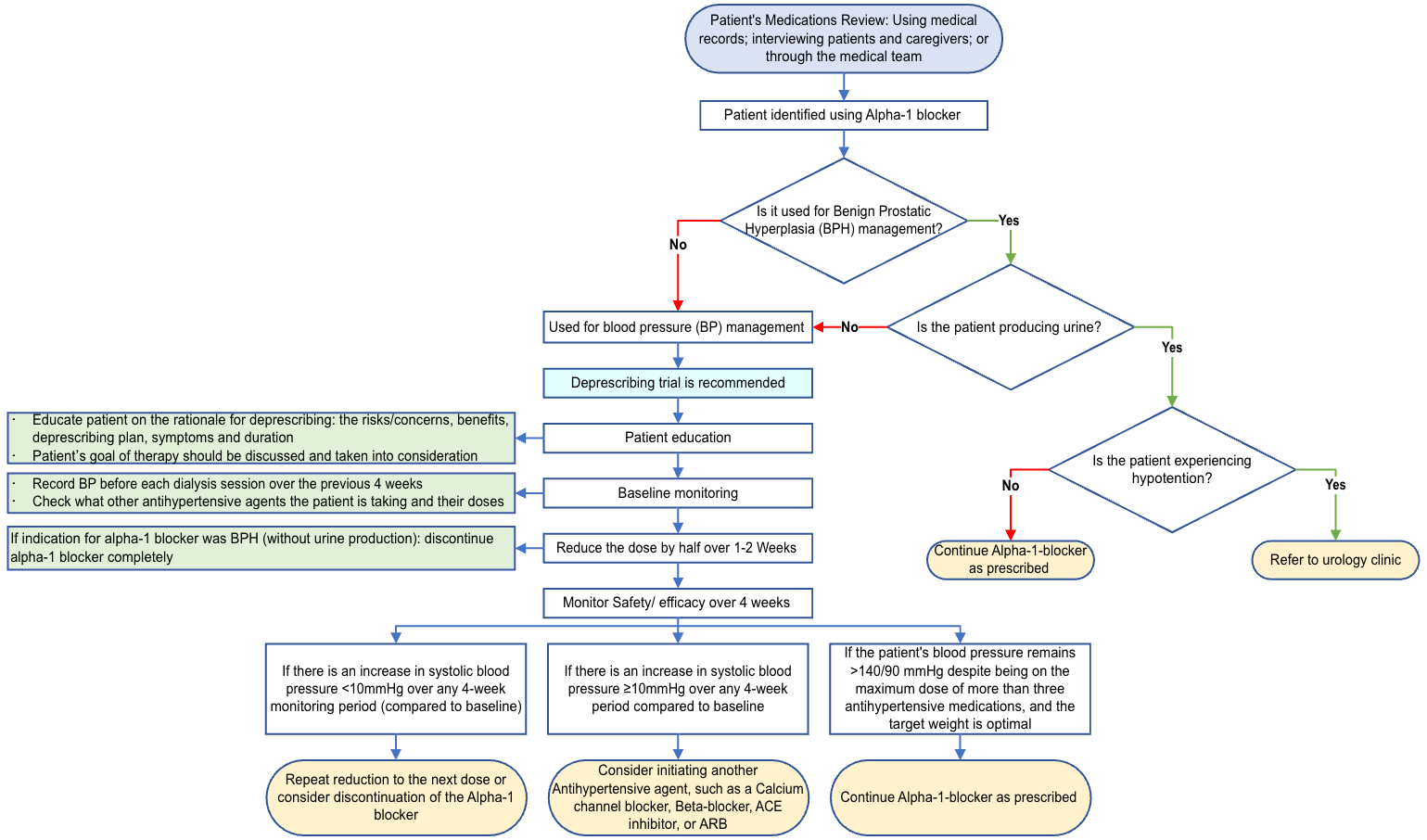


**References:**

- Lefebvre, M.J., et al., *Development and validation of nine deprescribing algorithms for patients on hemodialysis to decrease polypharmacy.* Canadian Journal of Kidney Health and Disease, 2020. **7**: p. 2054358120968674.
- Gerardi, S., et al., *Implementation of targeted deprescribing of potentially inappropriate medications in patients on hemodialysis.* American Journal of Health-System Pharmacy, 2022. **79**(Supplement_4): p. S128-S135.

## Statins


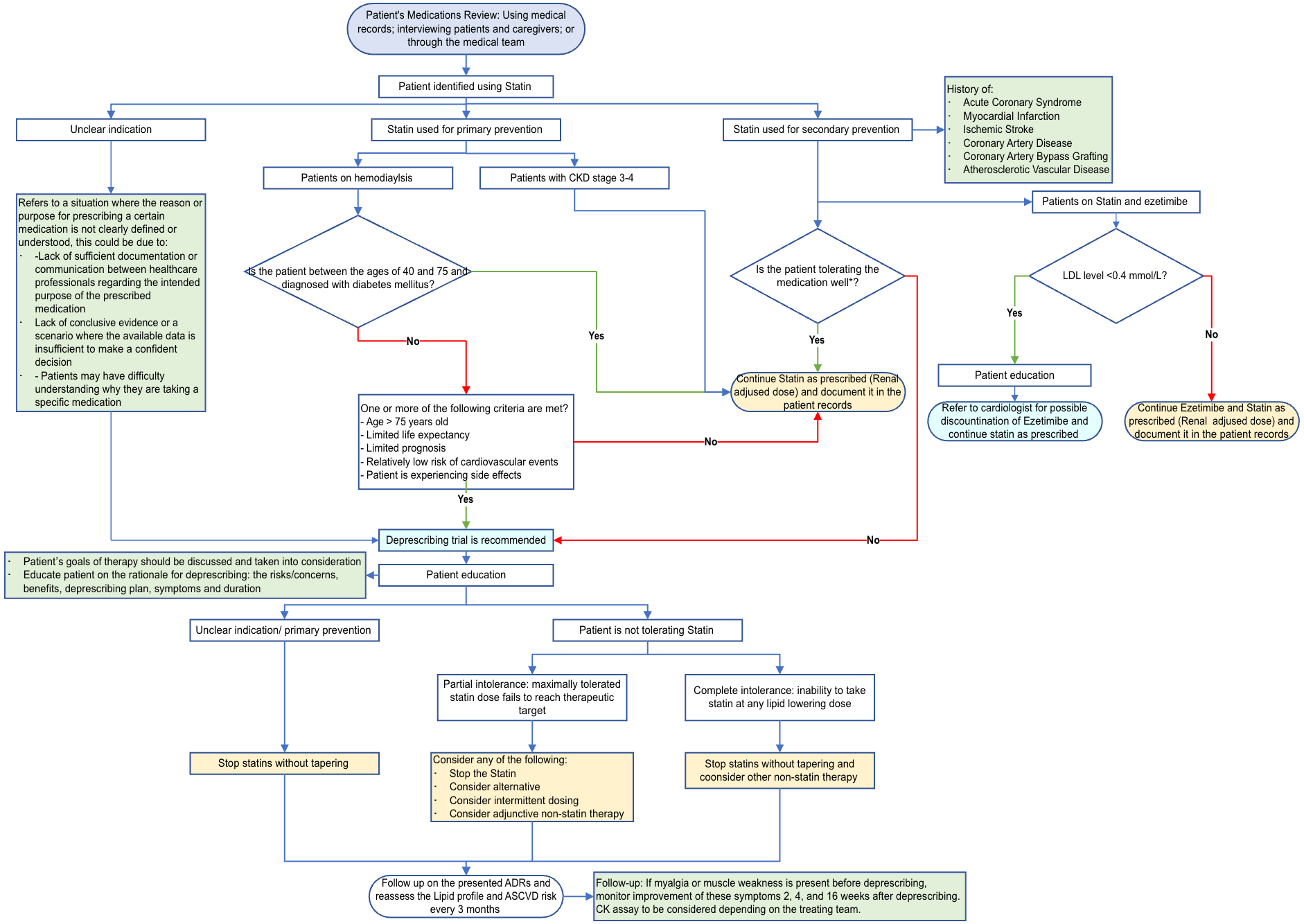


**References**:

- Lefebvre, M.J., et al., *Development and validation of nine deprescribing algorithms for patients on hemodialysis to decrease polypharmacy.* Canadian Journal of Kidney Health and Disease, 2020. **7**: p. 2054358120968674.
- Gerardi, S., et al., *Implementation of targeted deprescribing of potentially inappropriate medications in patients on hemodialysis.* American Journal of Health-System Pharmacy, 2022. **79**(Supplement_4): p. S128-S135.
- Cheung, A.K., et al., *KDIGO 2021 clinical practice guideline for the management of blood pressure in chronic kidney disease.* Kidney International, 2021. **99**(3): p. S1-S87.
- Rossing, P., et al., *KDIGO 2022 clinical practice guideline for diabetes management in chronic kidney disease.* Kidney international, 2022. **102**(5): p. S1-S127.
- Wanner, C. and M. Tonelli, *KDIGO Clinical Practice Guideline for Lipid Management in CKD: summary of recommendation statements and clinical approach to the patient.* Kidney international, 2014. **85**(6): p. 1303-1309.
- Societies, E.N.C., et al., *2019 ESC/EAS guidelines for the management of dyslipidaemias: lipid modification to reduce cardiovascular risk.* 2019.
- Primary Health Tasmania, *A Guide to deprescribing Statins.* 2022.

## Proton Pump Inhibitors (PPIs)


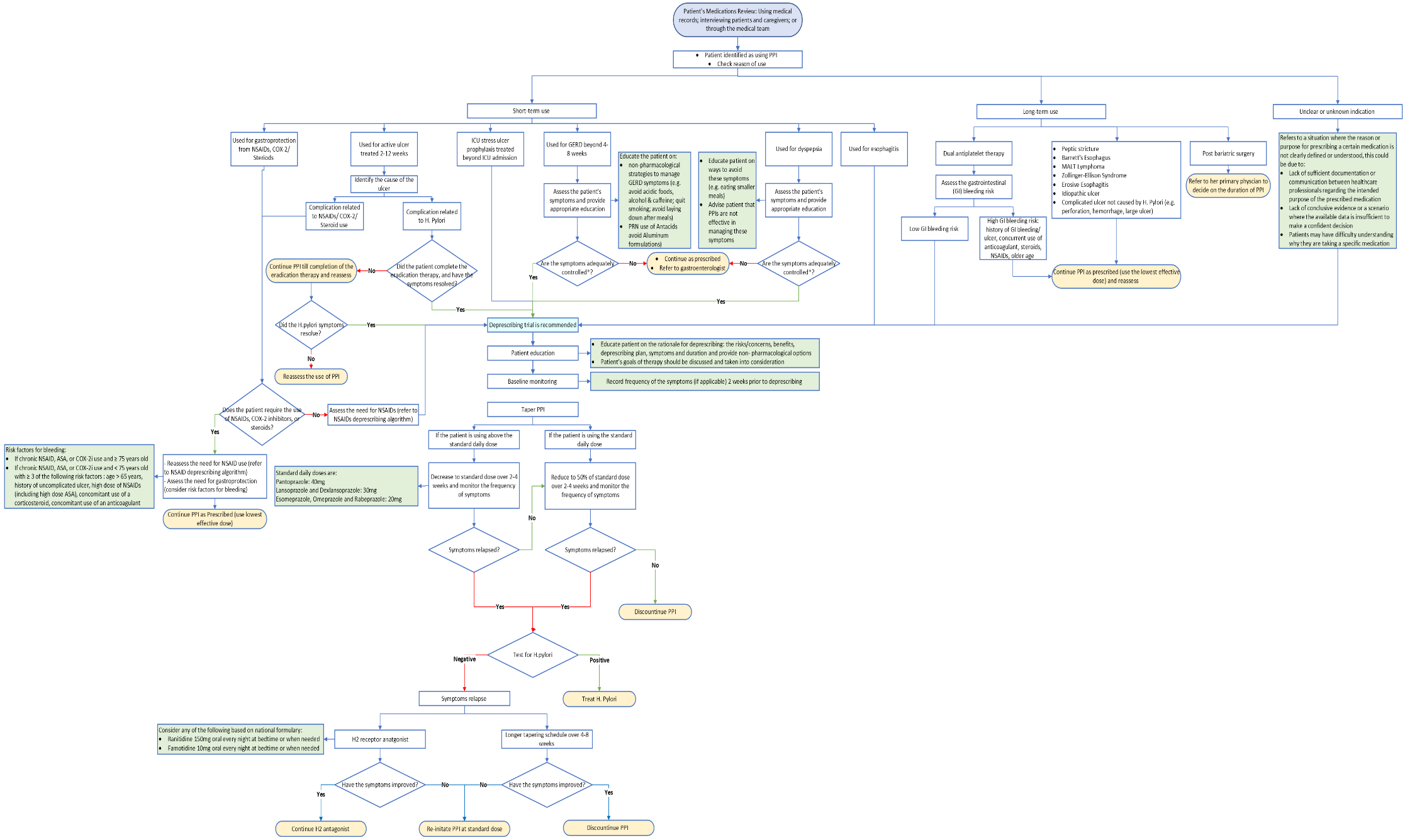


**References:**

- Lefebvre MJ, Ng PC, Desjarlais A, McCann D, Waldvogel B, Tonelli M, et al. Development and validation of nine deprescribing algorithms for patients on hemodialysis to decrease polypharmacy. *CanadianJournal* *of* *Kidney* *Health* *and* *Disease*. 2020 Jan;7:205435812096867. doi:10.1177/2054358120968674
- Gerardi S, Sperlea D, Levy SO-L, Bondurant-David K, Dang S, David P-M, et al. Implementation of targeted deprescribing of potentially inappropriate medications in patients on hemodialysis. American Journal of Health-System Pharmacy. 2022 Jul 26;79(Supplement_4). doi:10.1093/ajhp/zxac190
- Farrell B, Pottie K, Thompson W, Boghossian T, Pizzola L, Rashid FJ, et al. Deprescribing proton pump inhibitors. Evidence-based clinical practice guideline. Can Fam Physician 2017;63:354-64 (Eng), e253-65 (Fr).
- https://www.primaryhealthtas.com.au/wp-content/uploads/2023/03/A-guide-to-deprescribing-proton-pump-inhibitors.pdf

## Benzodiazepine and Z-drugs


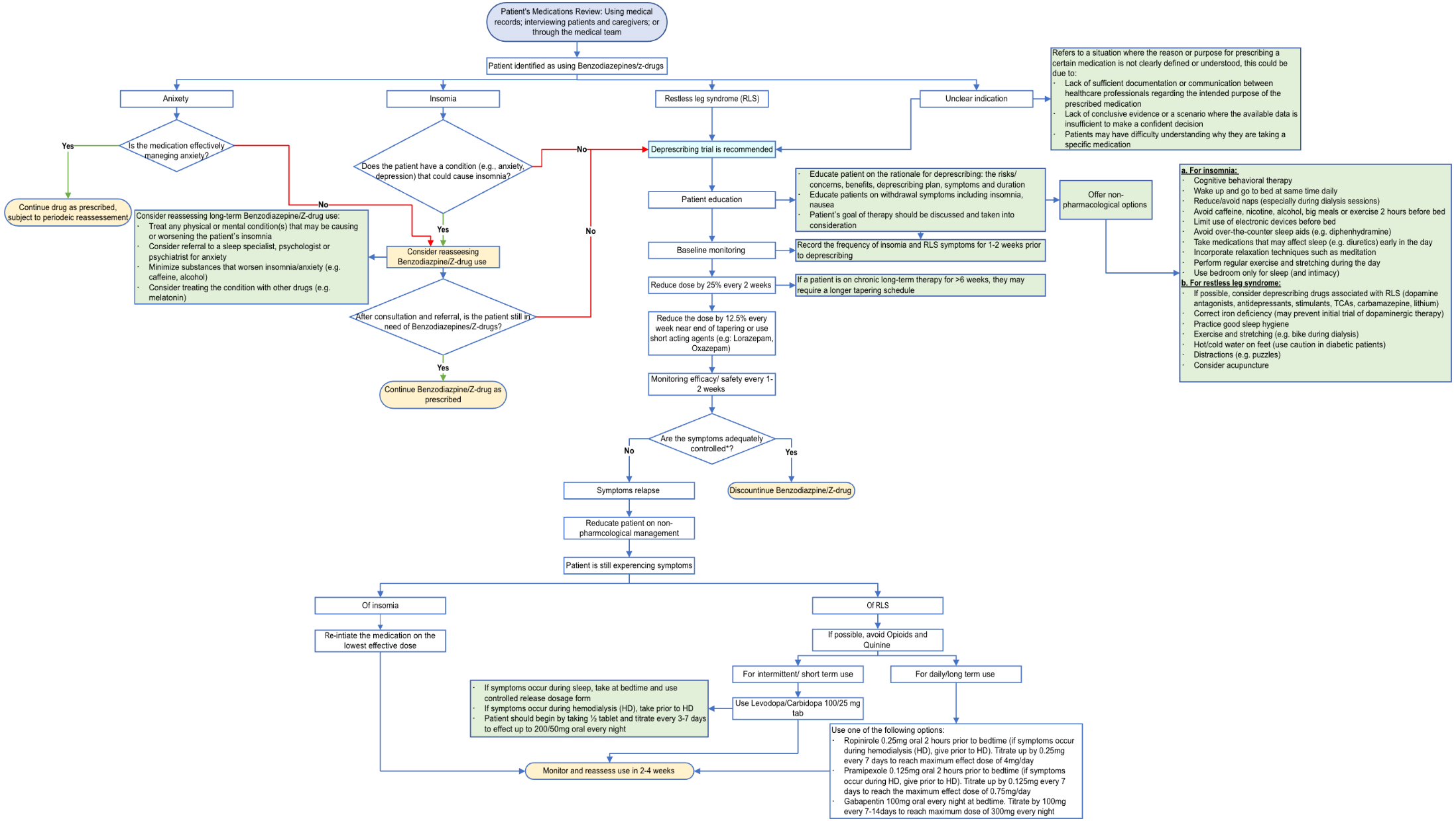


**References:**

- Lefebvre MJ, Ng PC, Desjarlais A, McCann D, Waldvogel B, Tonelli M, et al. Development and validation of nine deprescribing algorithms for patients on hemodialysis to decrease polypharmacy. *CanadianJournal* *of* *Kidney* *Health* *and* *Disease*. 2020 Jan;7:205435812096867. doi:10.1177/2054358120968674
- Gerardi S, Sperlea D, Levy SO-L, Bondurant-David K, Dang S, David P-M, et al. Implementation of targeted deprescribing of potentially inappropriate medications in patients on hemodialysis. American Journal of Health-System Pharmacy. 2022 Jul 26;79(Supplement_4). doi:10.1093/ajhp/zxac190
- https://www.primaryhealthtas.com.au/wp-content/uploads/2023/03/A-guide-to-deprescribing-benzodiazepines.pdf

## Urate-lowering agents
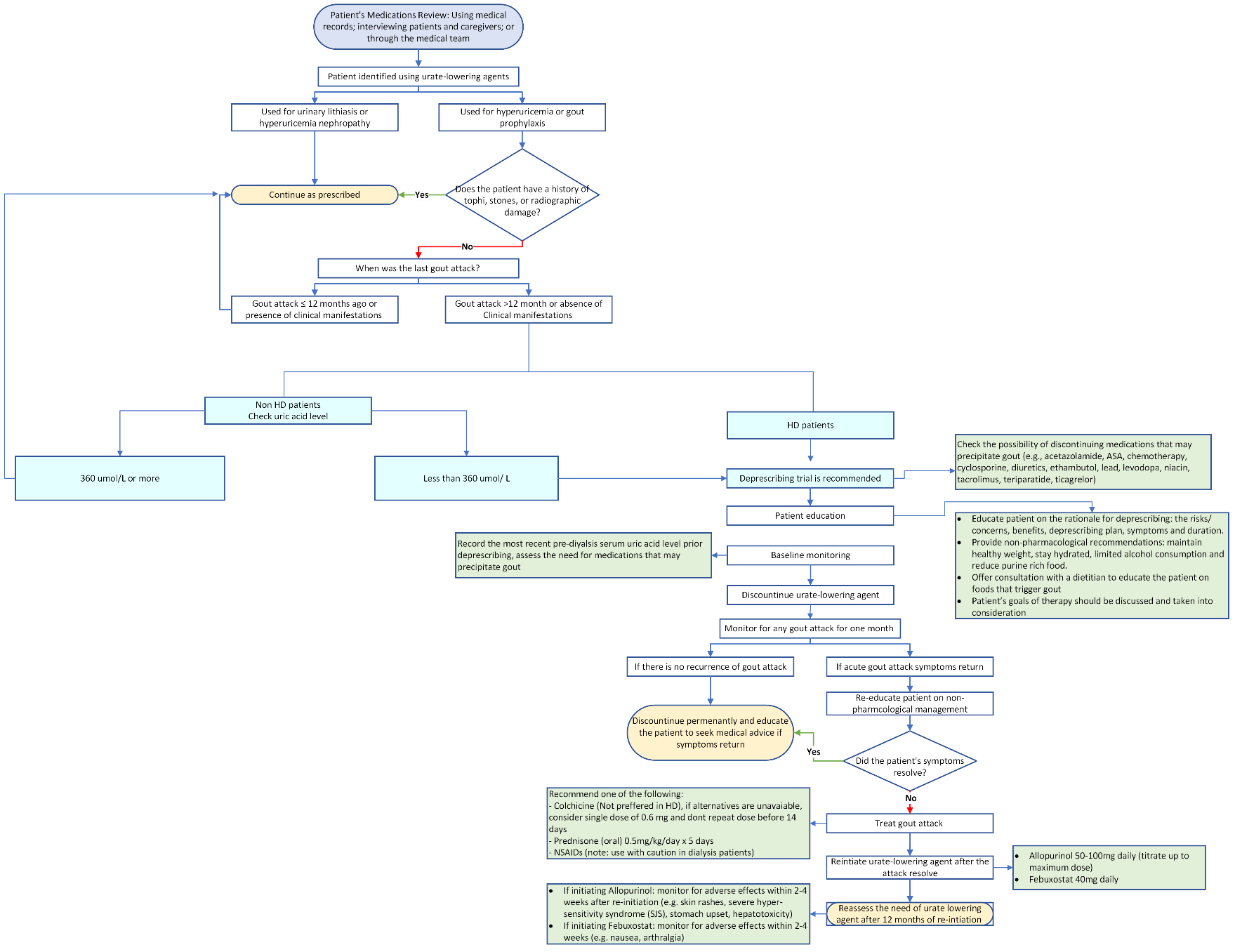


**References:**

- Lefebvre MJ, Ng PC, Desjarlais A, McCann D, Waldvogel B, Tonelli M, et al. Development and validation of nine deprescribing algorithms for patients on hemodialysis to decrease polypharmacy. *CanadianJournal* *of* *Kidney* *Health* *and* *Disease*. 2020 Jan;7:205435812096867. doi:10.1177/2054358120968674
- Gerardi S, Sperlea D, Levy SO-L, Bondurant-David K, Dang S, David P-M, et al. Implementation of targeted deprescribing of potentially inappropriate medications in patients on hemodialysis. American Journal of Health-System Pharmacy. 2022 Jul 26;79(Supplement_4). doi:10.1093/ajhp/zxac190
- Levin A, Ahmed SB, Carrero JJ, Foster B, Francis A, Hall RK, Herrington WG, Hill G, Inker LA, Kazancıoğlu R, Lamb E. Executive summary of the KDIGO 2024 Clinical Practice Guideline for the Evaluation and Management of Chronic Kidney Disease: known knowns and known unknowns. Kidney international. 2024 Apr 1;105(4):684-701.
- https://www.primaryhealthtas.com.au/wp-content/uploads/2023/03/A-guide-to-deprescribing-allopurinol.pdf

## Prokinetic agents


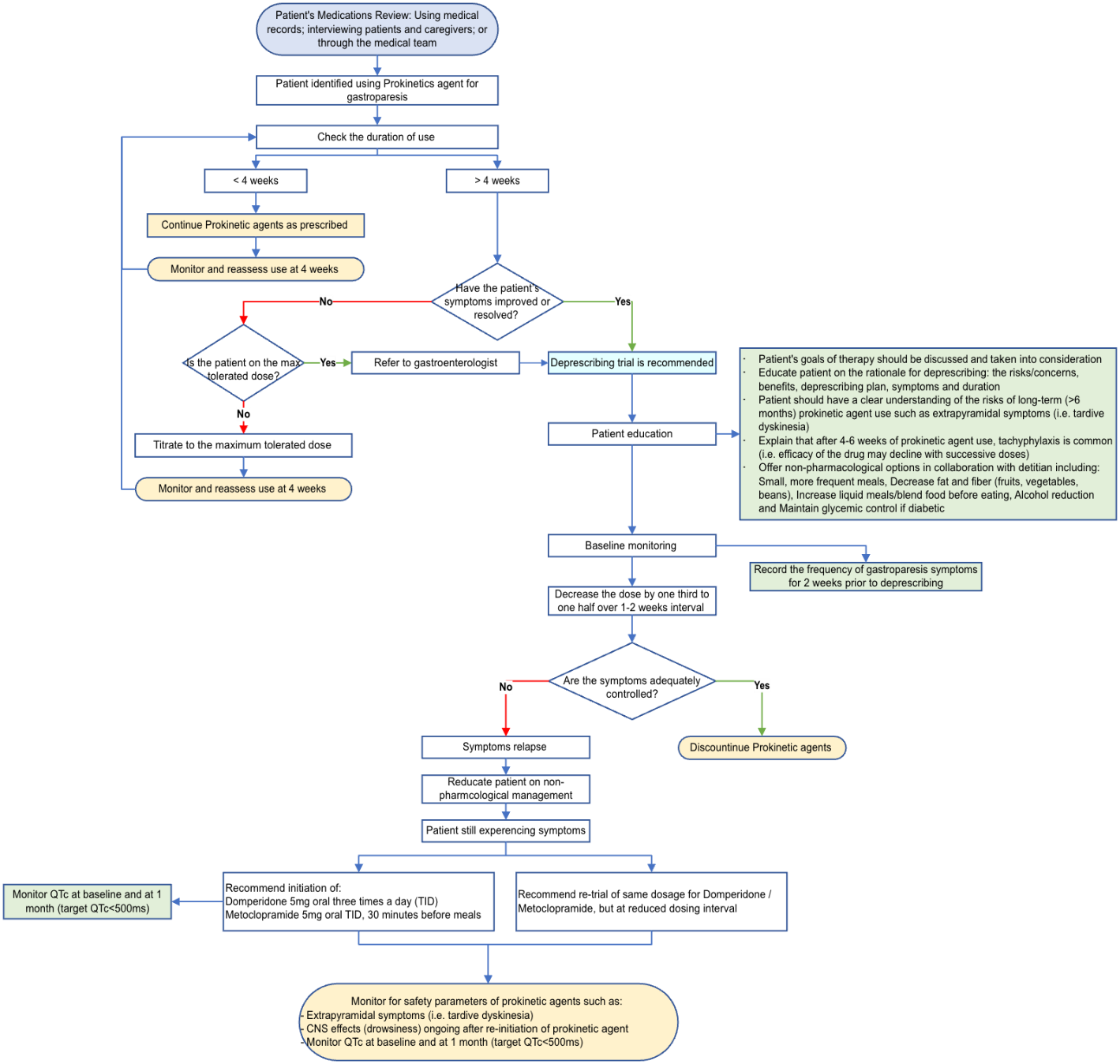


**References:**

- Lefebvre MJ, Ng PC, Desjarlais A, McCann D, Waldvogel B, Tonelli M, et al. Development and validation of nine deprescribing algorithms for patients on hemodialysis to decrease polypharmacy. *CanadianJournal* *of* *Kidney* *Health* *and* *Disease*. 2020 Jan;7:205435812096867. doi:10.1177/2054358120968674

## Gabapentinoids


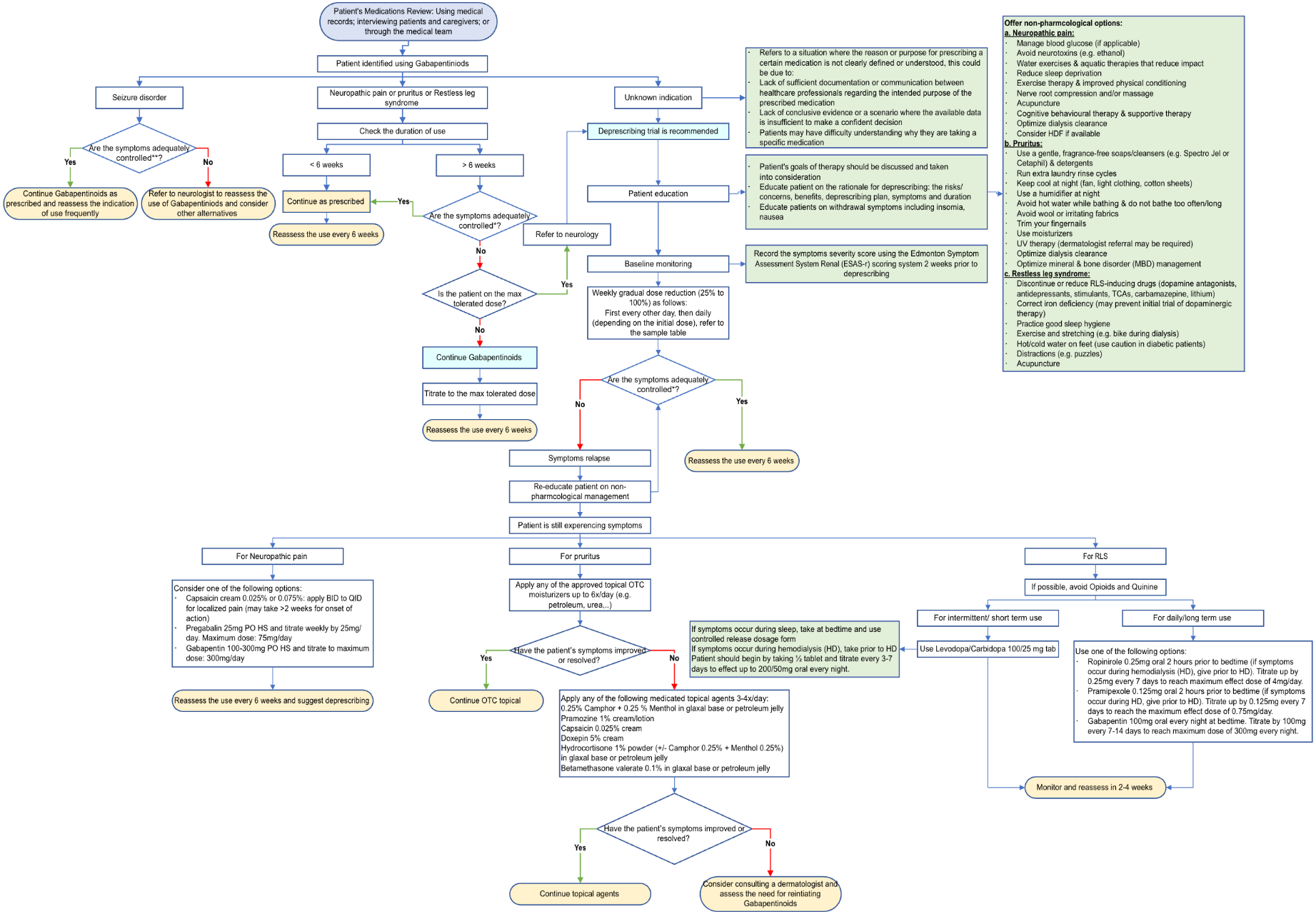


**References:**

- Lefebvre MJ, Ng PC, Desjarlais A, McCann D, Waldvogel B, Tonelli M, et al. Development and validation of nine deprescribing algorithms for patients on hemodialysis to decrease polypharmacy. *CanadianJournal* *of* *Kidney* *Health* *and* *Disease*. 2020 Jan;7:205435812096867. doi:10.1177/2054358120968674
- https://www.primaryhealthtas.com.au/wp-content/uploads/2023/03/A-guide-to-deprescribing-gabapentinoids.pdf

## Oral Anti-hyperglycemic


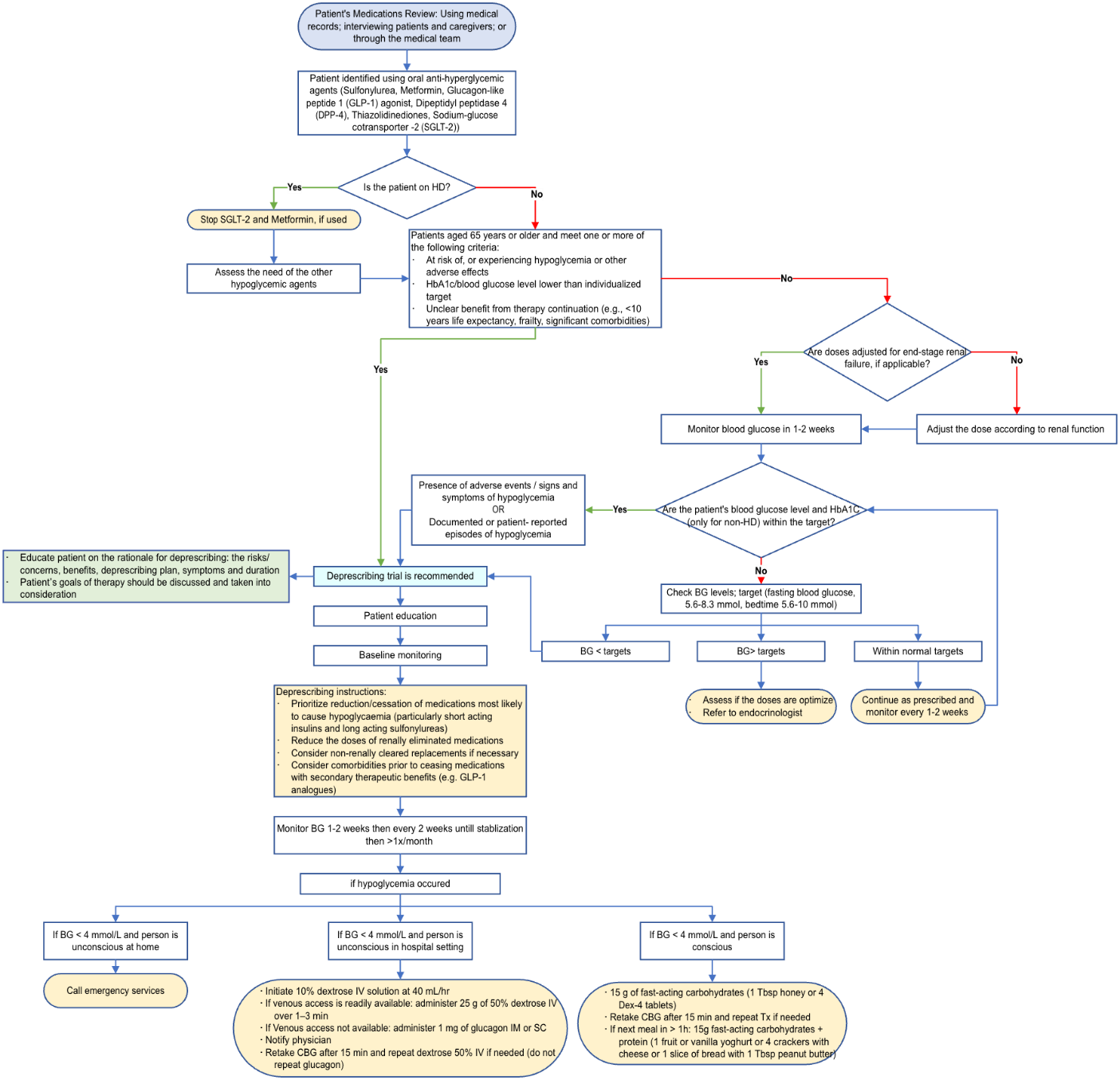


**References:**

- Gerardi S, Sperlea D, Levy SO-L, Bondurant-David K, Dang S, David P-M, et al. Implementation of targeted deprescribing of potentially inappropriate medications in patients on hemodialysis. American Journal of Health-System Pharmacy. 2022 Jul 26;79(Supplement_4). doi:10.1093/ajhp/zxac190
- https://www.primaryhealthtas.com.au/wp-content/uploads/2023/03/A-guide-to-deprescribing-antihyperglycaemics.pdf

## Antihypertensive agents


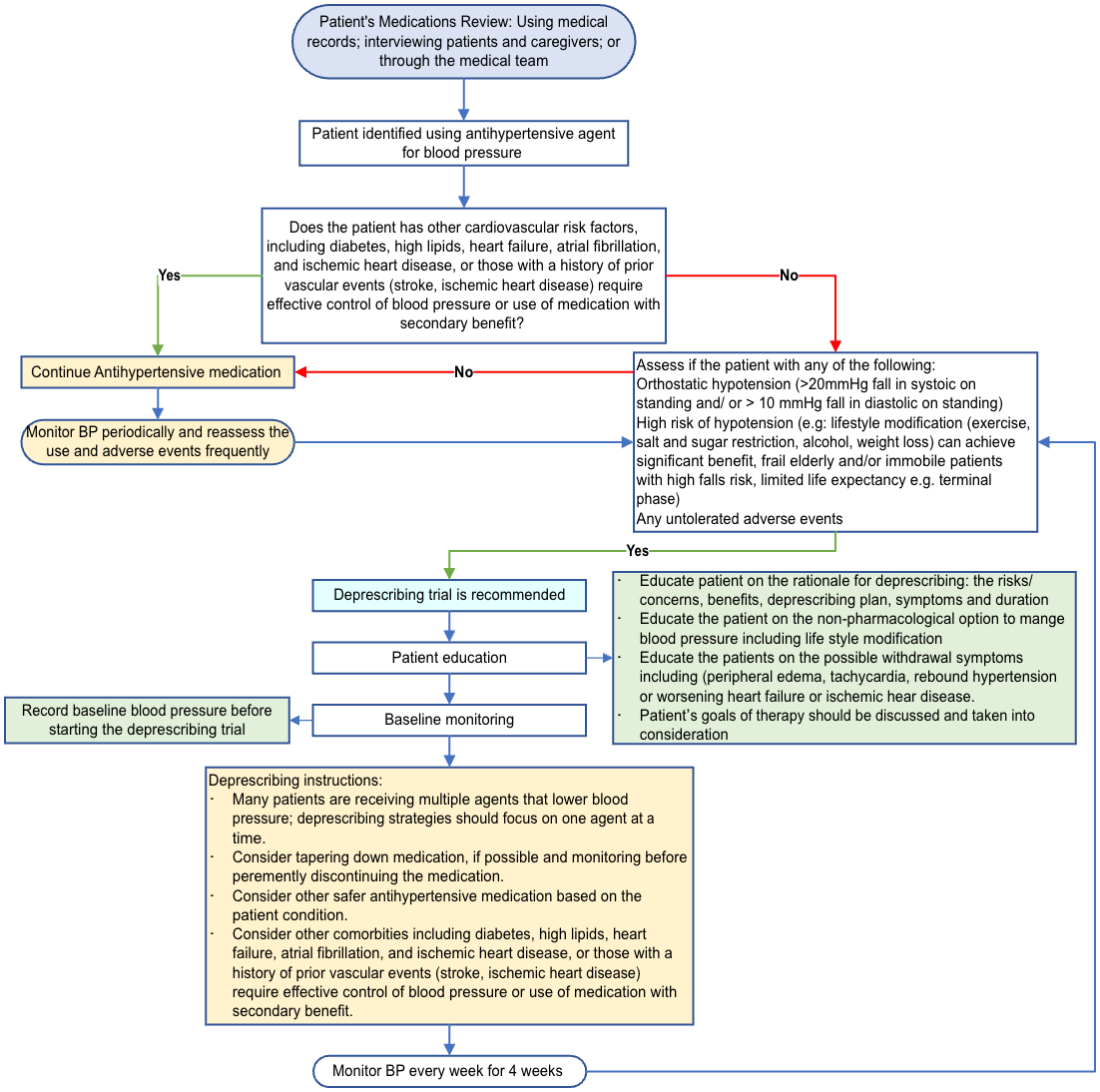


**References:**

- McEvoy JW, McCarthy CP, Bruno RM, Brouwers S, Canavan MD, Ceconi C, Christodorescu RM, Daskalopoulou SS, Ferro CJ, Gerdts E, Hanssen H. 2024 ESC Guidelines for the management of elevated blood pressure and hypertension: Developed by the task force on the management of elevated blood pressure and hypertension of the European Society of Cardiology (ESC) and endorsed by the European Society of Endocrinology (ESE) and the European Stroke Organisation (ESO). European heart journal. 2024 Oct 7;45(38):3912-4018.
- https://www.primaryhealthtas.com.au/wp-content/uploads/2023/03/A-guide-to-deprescribing-antihypertensives.pdf

## Anticoagulants


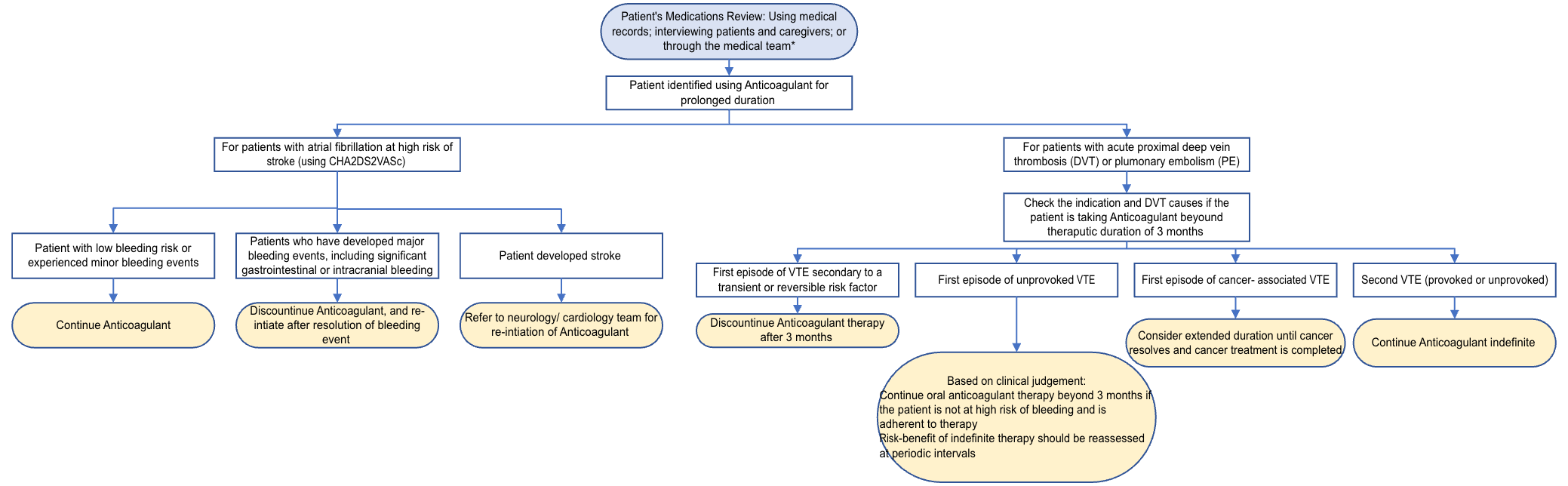


**References:**

- Čihák R, Haman L, Táborský M. 2016 ESC Guidelines for the management of atrial fibrillation developed in collaboration with EACTS: summary of the document prepared by the Czech Society of Cardiology. Cor et vasa. 2016 Dec 1;58(6):e636-83
- January CT, Wann LS, Alpert JS, Calkins H, Cigarroa JE, Cleveland Jr JC, Conti JB, Ellinor PT, Ezekowitz MD, Field ME, Murray KT. 2014 AHA/ACC/HRS guideline for the management of patients with atrial fibrillation: executive summary: a report of the American College of Cardiology/American Heart Association Task Force on practice guidelines and the Heart Rhythm Society. Circulation. 2014 Dec 2;130(23):2071-104.
- Van Gelder IC, Rienstra M, Bunting KV, Casado-Arroyo R, Caso V, Crijns HJ, De Potter TJ, Dwight J, Guasti L, Hanke T, Jaarsma T. 2024 ESC Guidelines for the management of atrial fibrillation developed in collaboration with the European Association for Cardio-Thoracic Surgery (EACTS) Developed by the task force for the management of atrial fibrillation of the European Society of Cardiology (ESC), with the special contribution of the European Heart Rhythm Association (EHRA) of the ESC. Endorsed by the European Stroke Organisation (ESO). European Heart Journal. 2024 Aug 30:ehae176.
- <https://www.primaryhealthtas.com.au/wp-content/uploads/2023/03/A-guide-to-deprescribing-anticoagulants.pdf>

## Aspirin


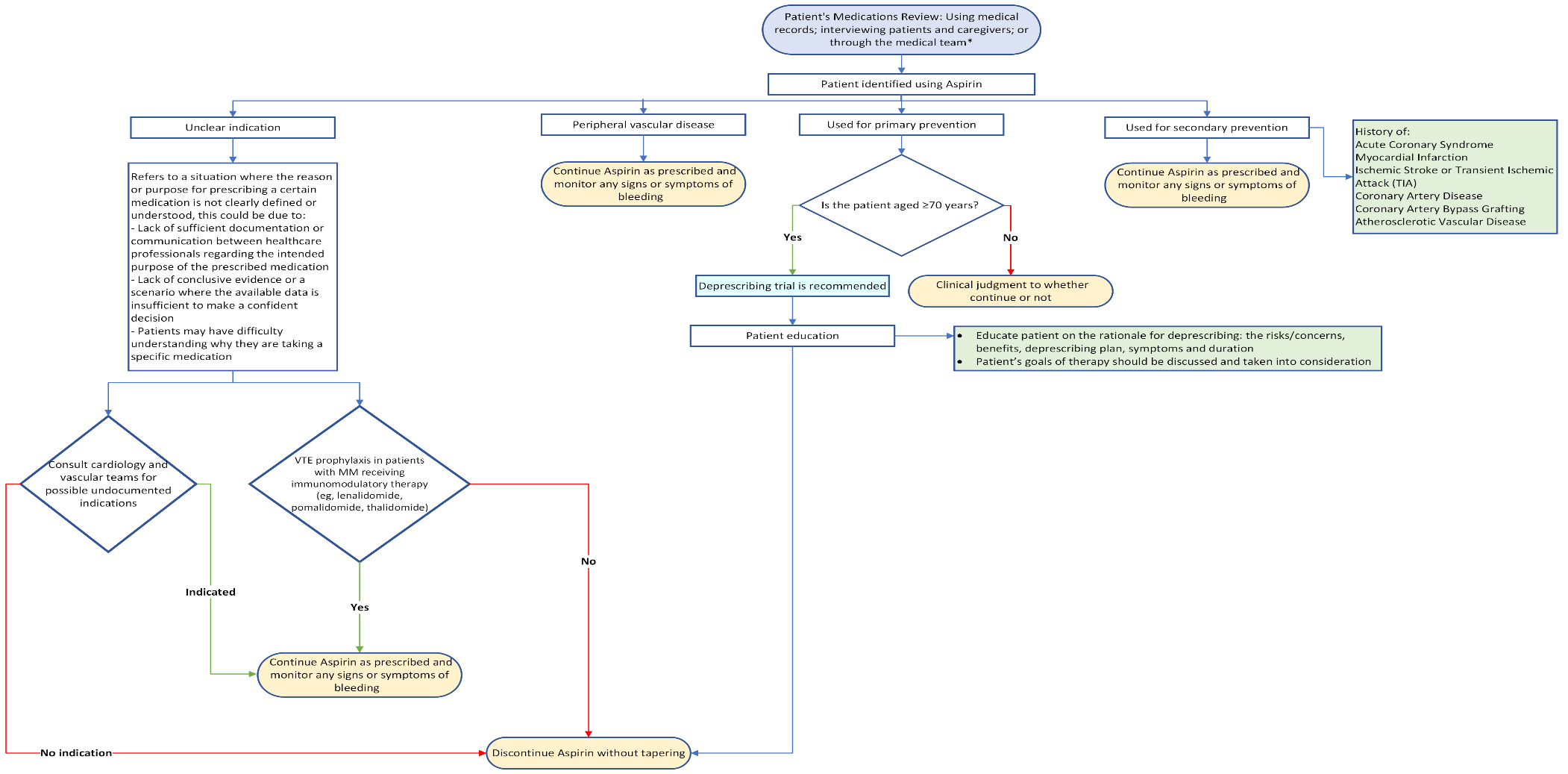


*****Please confirm patient adherence and ensure that he/she is taking the medication as prescribed before using the algorithm.

VTE: Venous thromboembolism, MM: Multiple myeloma.

**References:**

- Gerardi S, Sperlea D, Levy SO-L, Bondurant-David K, Dang S, David P-M, et al. Implementation of targeted deprescribing of potentially inappropriate medications in patients on hemodialysis. American Journal of Health-System Pharmacy. 2022 Jul 26;79(Supplement_4). doi:10.1093/ajhp/zxac190.

## Antiplatelets


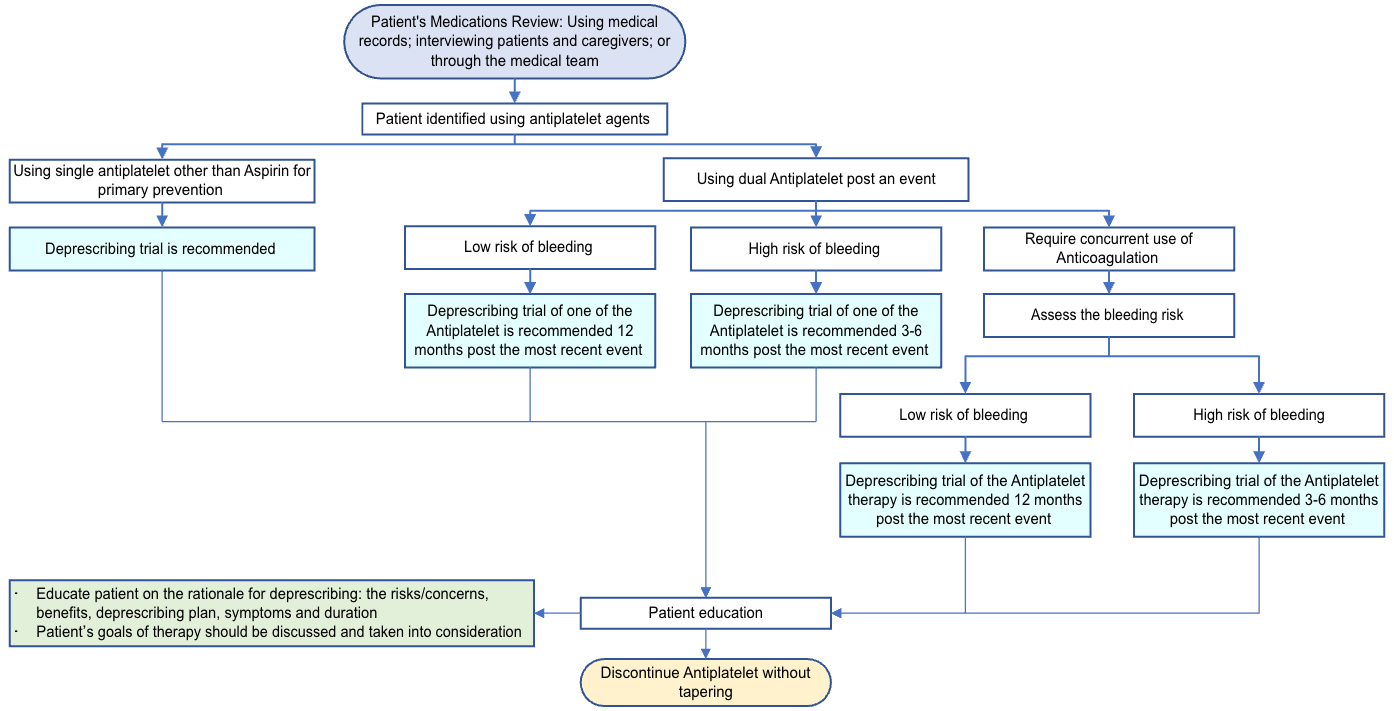
**References:**

- Gerardi S, Sperlea D, Levy SO-L, Bondurant-David K, Dang S, David P-M, et al. Implementation of targeted deprescribing of potentially inappropriate medications in patients on hemodialysis. American Journal of Health-System Pharmacy. 2022 Jul 26;79(Supplement_4). doi:10.1093/ajhp/zxac190
- https://www.primaryhealthtas.com.au/wp-content/uploads/2023/03/A-guide-to-deprescribing-antiplatelets.pdf

## Long-acting Nitrates


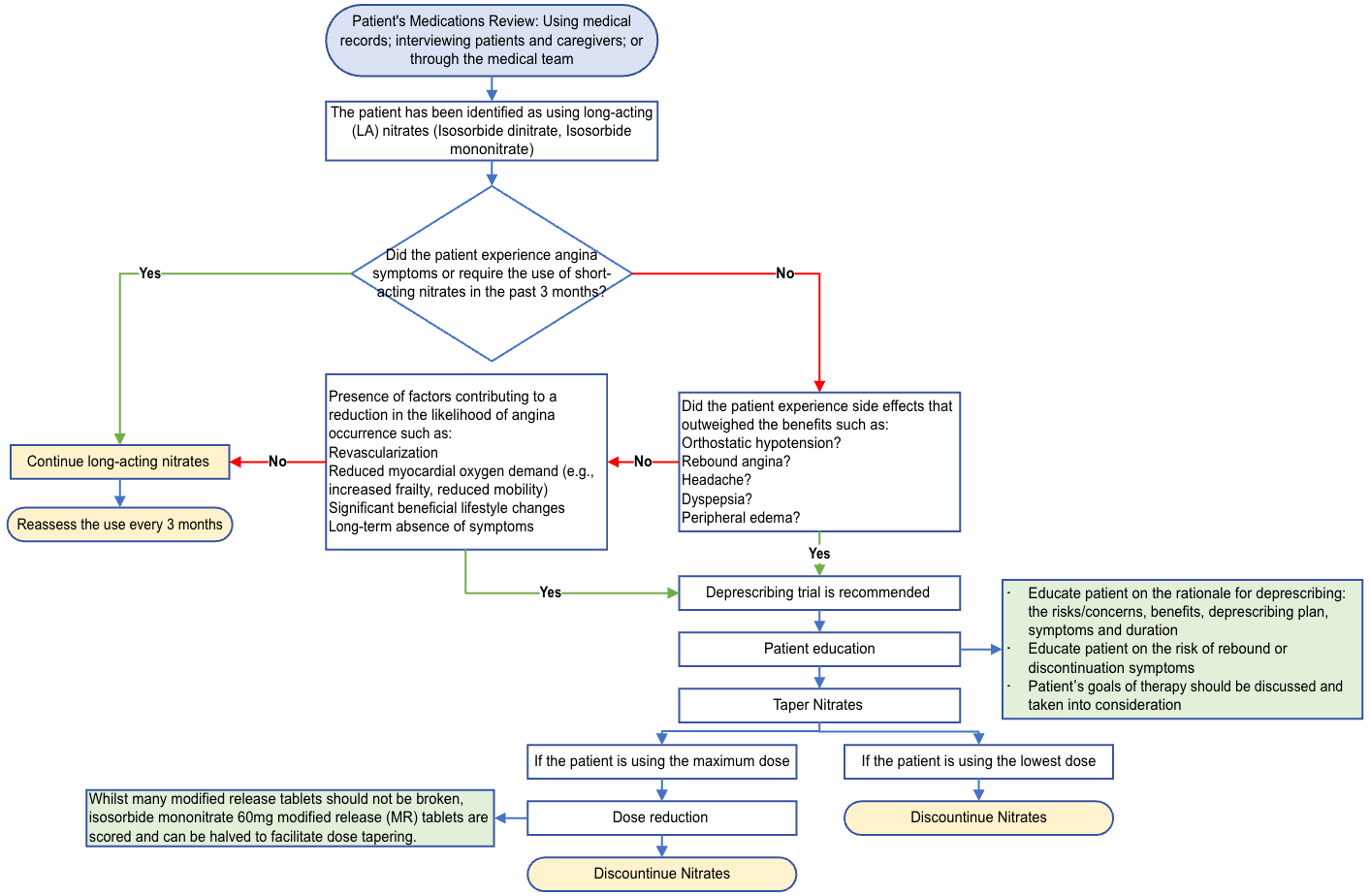


**References:**

- https://www.primaryhealthtas.com.au/wp-content/uploads/2023/03/A-guide-to-deprescribing-long-acting-nitrates.pdf

## NSAIDs


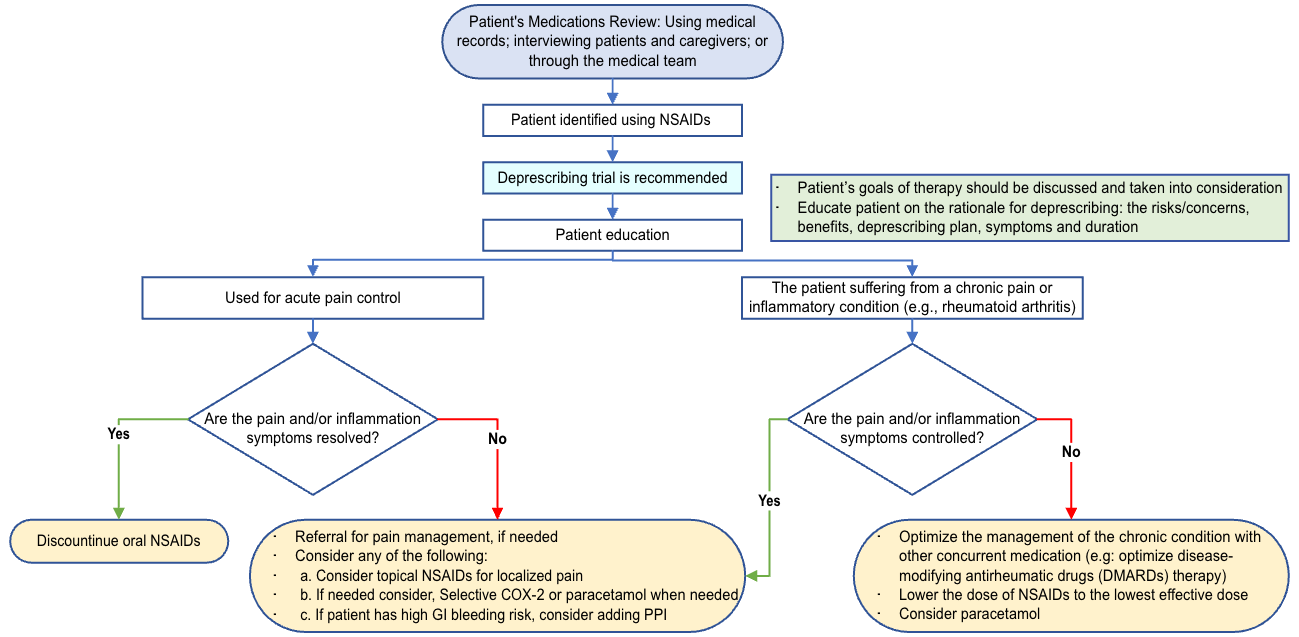


**References:**

- https://www.primaryhealthtas.com.au/wp-content/uploads/2023/03/A-guide-to-deprescribing-non-steroidal-anti-inflammatory-drugs.pdf

## Anticholinergic agents


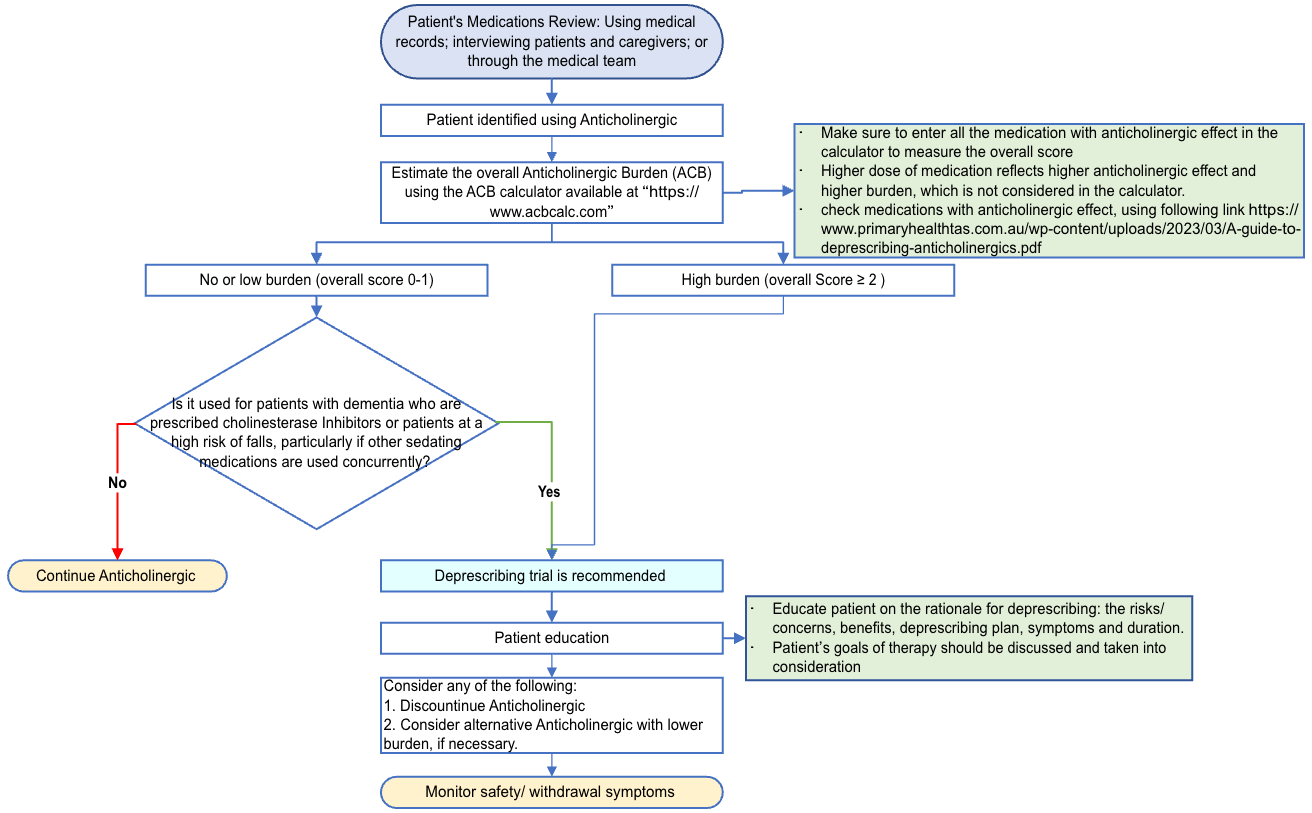


**References:**

- <https://www.primaryhealthtas.com.au/wp-content/uploads/2023/03/A-guide-to-deprescribing-anticholinergics.pdf>
- [https://www.Acbcalc.Com](https://www.acbcalc.com)]

## Antipsychotic agents


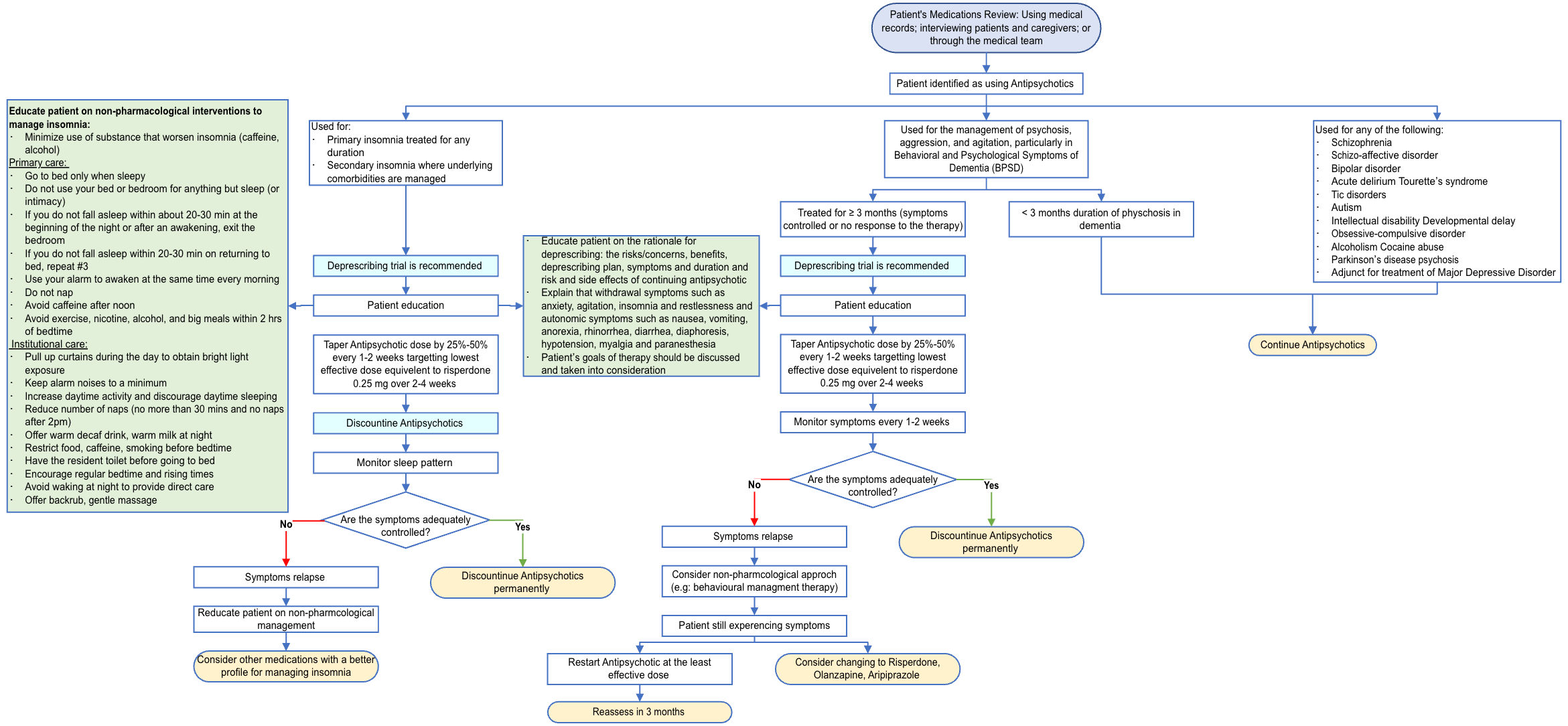


**References:**

- Bjerre LM, Farrell B, Hogel M, Graham L, Lemay G, McCarthy L, Raman-Wilms L, Rojas-Fernandez C, Sinha S, Thompson W, Welch V. Deprescribing antipsychotics for behavioural and psychological symptoms of dementia and insomnia: evidence-based clinical practice guideline. Canadian Family Physician. 2018 Jan 1;64(1):17-27.
- <https://www.primaryhealthtas.com.au/wp-content/uploads/2023/03/A-guide-to-deprescribing-antipsychotics.pdf>

# Resources:

1. Zidan, A. and A. Awaisu, *Inappropriate polypharmacy management versus deprescribing: A review on their relationship.* Basic & Clinical Pharmacology & Toxicology, 2024. **134**(1): p. 6-14.

2. Reeve, E., et al., *Review of deprescribing processes and development of an evidence‐based, patient‐centred deprescribing process.* British journal of clinical pharmacology, 2014. **78**(4): p. 738-747.

3. Mohottige, D., H.J. Manley, and R.K. Hall, *Less is more: deprescribing medications in older adults with kidney disease: a review.* Kidney360, 2021. **2**(9): p. 1510-1522.

4. Lefebvre, M.J., et al., *Development and validation of nine deprescribing algorithms for patients on hemodialysis to decrease polypharmacy.* Canadian Journal of Kidney Health and Disease, 2020. **7**: p. 2054358120968674.

5. Radcliffe, E., et al., *What makes a multidisciplinary medication review and deprescribing intervention for older people work well in primary care? A realist review and synthesis.* BMC geriatrics, 2023. **23**(1): p. 591.

6. Jones, K.F., et al., *Patient-directed education to promote deprescribing: a nonrandomized clinical trial.* JAMA Internal Medicine, 2024. **184**(11): p. 1339-1346.

7. Hanlon, J.T., et al., *A method for assessing drug therapy appropriateness.* Journal of clinical epidemiology, 1992. **45**(10): p. 1045-1051.

8. West, L.M., M. Cordina, and S. Cunningham, *Clinical pharmacist evaluation of medication inappropriateness in the emergency department of a teaching hospital in Malta.* Pharmacy practice, 2012. **10**(4): p. 181.
